# Therapy‐Induced ECM Remodeling Creates a Transient Immune Barrier in Residual Melanoma

**DOI:** 10.1002/advs.202508451

**Published:** 2025-08-22

**Authors:** Chia‐Hsin Hsu, Jingyi Chen, Keng‐Jung Lee, Leanne R. Donahue, Danielle Kacaj, Sean P. McDonough, Andrew C. White

**Affiliations:** ^1^ Department of Biomedical Sciences Cornell University Ithaca NY 14853 USA; ^2^ Department of Biomedical Engineering Carnegie Mellon University Pittsburgh PA 15205 USA; ^3^ Department of Population Medicine and Diagnostic Sciences Cornell University Ithaca NY 14853 USA

**Keywords:** BRAF/MEK inhibitors, CD8+ T cells, collagen, ECM, melanoma, residual disease, therapy resistance

## Abstract

Targeted therapies reshape the tumor not only by eliminating malignant cells but also by altering the stromal and immunologic adaptations that emerge during treatment, which remain incompletely defined. Here, extracellular matrix (ECM) remodeling is identified as a key driver of immune exclusion during the residual disease phase—a transient, therapy‐tolerant state that precedes overt resistance. Using an immune‐competent melanoma model and temporal transcriptomic profiling of tumor cells and fibroblasts, a coordinated induction of ECM‐related genes, particularly collagen, is uncovered during the development of residual disease. This remodeling creates a physical barrier that spatially excludes CD8⁺ T cells from residual tumor niches, compromising immune surveillance. Human melanoma datasets validate increased ECM gene expression and show an inverse correlation between collagen and cytotoxic T lymphocyte infiltration, as well as patient survival. Strikingly, pharmacologic inhibition of collagen deposition, administered at the point of maximal tumor regression, restores CD8⁺ T cell infiltration and delays resistance in a CD8⁺ T cell–dependent manner. These findings define residual disease as a therapeutically actionable stromal state and demonstrate that ECM modulation can overcome immune exclusion, thereby improving the durability of targeted therapy responses.

## Graphical abstract

BRAF/MEK inhibition promotes extracellular matrix (ECM) accumulation and remodeling during therapy‐tolerant residual disease, limiting CD8⁺ T cell access to tumor cells. Targeting ECM accumulation restores CD8⁺ T cell infiltration, enhances their intratumoral distribution, and delays therapeutic resistance. These findings identify ECM remodeling as a critical barrier to antitumor immunity during targeted therapy in melanoma.

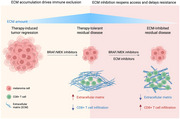

## 1 Introduction

Resistance to cancer therapies remains a major barrier to long‐term clinical success, particularly in solid tumors such as melanoma. While therapy resistance has been linked to both genetic and non‐genetic reprogramming within cancer cells, accumulating evidence highlights the tumor microenvironment (TME) as a critical, but less well‐characterized, contributor to this process.^[^ ^1^ ^]^ Tumor relapse is thought to originate from a population of drug‐tolerant persister cells that survive initial treatment, giving rise to minimal residual disease (MRD).^[^ ^2^ ^]^ These persister cells may arise through transcriptional and epigenetic mechanisms or emerge from pre‐existing heterogeneity.^[^ ^2^, ^3^, ^4^ ^]^ Additionally, recent work suggests that drug resistance emerges from these persisters in multiple independent trajectories, even among isogenic cell populations.^[^ ^5^ ^]^

In BRAF^V600^‐mutant melanoma, treatment with combined BRAF and MEK inhibitors (BRAF/MEKi) yields high initial response rates and is a standard‐of‐care therapy.^[^ ^6^ ^]^ However, resistance inevitably develops in nearly all patients, with a median duration of response of only 10.5 months.^[^ ^7^, ^8^ ^]^ Most strategies to counteract resistance have focused on cell‐intrinsic mechanisms, particularly MAPK pathway reactivation, yet no such approaches have achieved clinical approval. Meanwhile, the role of the evolving TME during the development of resistance, especially during the critical but transient MRD state, remains underexplored and poorly understood.

Emerging evidence indicates that the TME can be reprogrammed by targeted therapy and may actively support drug‐tolerant states.^[^ ^9^ ^]^ While BRAF/MEKi can promote anti‐tumor immune responses,^[^ ^10^, ^11^, ^12^ ^]^ how the TME changes during tumor regression and residual disease, and whether these changes generate barriers to effective immune surveillance, remains largely undefined. In particular, the potential contribution of therapy‐induced extracellular matrix (ECM) remodeling to immune exclusion and drug resistance has not been investigated in the context of MRD.

In this study, we address this critical knowledge gap by investigating the dynamic evolution of the TME during the transition from tumor regression to residual disease in an immunocompetent mouse model of BRAF‐mutant melanoma. Using temporal transcriptomic profiling of both melanoma cells and fibroblasts, we identify a coordinated upregulation of ECM‐related genes, notably collagen, during this transitional phase. We find that increased collagen deposition leads to physical exclusion of CD8⁺ T cells from residual tumors, impairing immune access and surveillance. Therapeutically, we show that pharmacologic inhibition of ECM organization, administered at the point of minimal tumor burden, restores CD8⁺ T cell infiltration and delays the emergence of BRAF/MEKi resistance in a CD8⁺ T cell–dependent manner. Consistent with our preclinical findings, melanoma patient datasets reveal increased ECM gene expression during treatment and an inverse relationship between collagen expression and CD8⁺ T cell infiltration.

Together, these findings fill a critical gap in our understanding of the TME during residual disease, identifying ECM remodeling as a modifiable barrier that restricts immune access and contributes to therapeutic failure. Targeting the ECM changes during this transitional window offers a promising strategy to enhance the durability of targeted therapies in melanoma. More broadly, our work positions residual disease as a tractable immunosuppressive niche, suggesting that integrating stromal modulation into therapy regimens may redefine how immune accessibility is restored during cancer treatment.

## 2 Results

### 2.1 BRAF/MEKi‐Tolerant Melanomas Show Distinct Gene Expression Profiles Compared to Regressing Tumors

**Figure 1:**
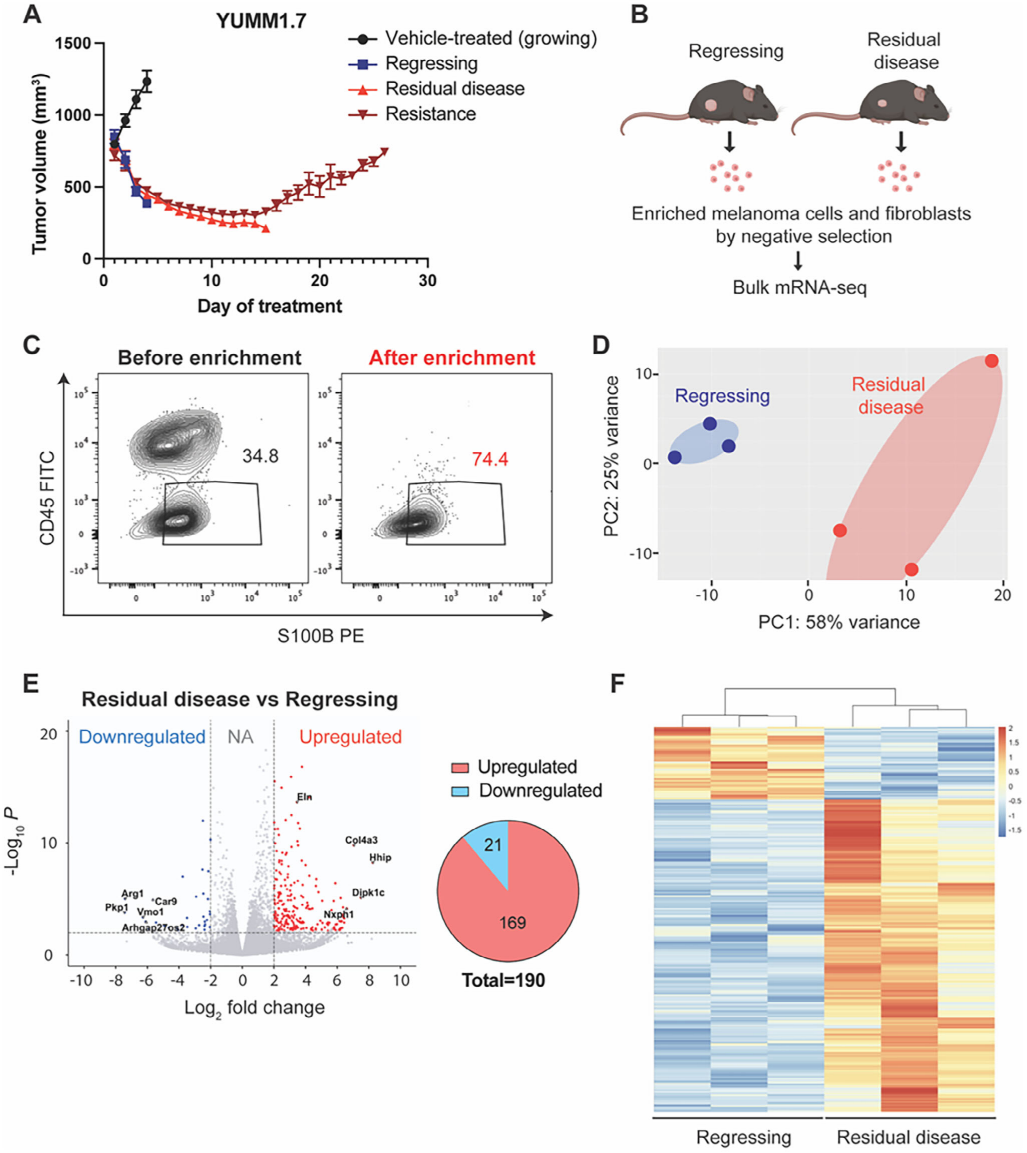
On‐therapy BRAF/MEKi‐tolerant tumors show distinct gene expression profiles compared to on‐therapy regressing tumors. A) Tumor volume curves showing the response of YUMM1.7 tumors to BRAF/MEK inhibition. Mice were treated with Dabrafenib (25 mg kg^−1^) and Trametinib (0.15 mg kg^−1^) via daily oral gavage, beginning when tumors reached ≈700 mm^3^. Vehicle‐treated tumors are labeled as “Vehicle‐treated (growing),” while treated tumors are categorized into regressing (3‐day BRAF/MEKi), residual disease (14‐day BRAF/MEKi), and resistant (continuous BRAF/MEKi treatment until tumors returned to pre‐treatment size) phases based on tumor dynamics. Tumor volumes were measured daily. Data are presented as mean ± SD; *n* = 5 tumors per group. B) Experimental scheme for bulk mRNA‐seq. Melanoma cells and cancer‐associated fibroblasts from regressing tumors and residual disease were enriched by negative selection followed by RNA isolation for bulk mRNA‐seq. C) Representative flow cytometry plot of melanoma cells gated on live cells before and after negative selection enrichment. Numbers on the plots represent the percent cells within each gate. CD45+ cells represent nucleated hematopoietic cells. S100B+ cells are melanoma cells. D) Principal component analysis plot indicating bulk mRNA‐seq sample clustering based on sample biological variability between regressing tumors and residual disease. E) Volcano plot showing differentially expressed genes of residual disease compared to regressing tumors (red: upregulated; blue: downregulated; ≥ 2‐fold change; *P_adj._* < 0.05). Venn diagram showing 190 differentially expressed genes in residual disease compared to regressing tumors. F) Heatmap demonstrating all differentially expressed genes between regressing tumors and residual disease. Rows represent genes, and columns represent individual tumor samples. Expression values are row‐wise Z‐score normalized (mean‐centered and scaled by standard deviation across samples for each gene). Red indicates higher expression and blue indicates lower expression relative to the gene's average expression. The result of the hierarchical clustering calculation is displayed as a dendrogram, showing the similarity between rows. Figure 1B was created with BioRender.com.

To model targeted therapy‐induced regression, the establishment of drug‐tolerant residual disease, drug resistance and tumor re‐growth, we transplanted Braf‐mutant murine melanoma cells (YUMM1.7) subcutaneously into C57BL/6J mice. Once tumors reached ≈700 mm^3^, we initiated daily oral gavage of Dabrafenib (a BRAF inhibitor, 25 mg kg^−1^) and Trametinib (a MEK inhibitor, 0.15 mg kg^−1^). Untreated controls received vehicle alone. Similar to previous studies, BRAF/MEKi induces a rapid tumor reduction within the first few days, which then leads to a plateau phase (drug‐tolerant, residual disease) after ≈2 weeks of treatment.^[^ ^2^, ^13^ ^]^ With continued BRAF/MEKi treatment, tumors develop full resistance and rebound to their initial size (i.e., ≈700mm^3^ in size) at ≈3 to 4 weeks of BRAF/MEKi administration (**Figure** **1**A; Figure S1A, Supporting Information). For clarity, we define “regressing tumors” as those undergoing active shrinkage during the early phase of BRAF/MEKi treatment (e.g., day 3), while “residual disease” refers to the stabilized, therapy‐tolerant tumor state observed after prolonged treatment (e.g., day 14), prior to overt resistance. Tumor relapse is thought to be seeded by a small population of residual, therapy‐tolerant cells that persist following initial tumor regression. These cells are critical therapeutic targets, as they represent the reservoir from which diverse resistance trajectories emerge. Furthermore, this residual disease phase is characterized by a lower tumor burden and limited cellular heterogeneity, providing a strategic window for therapeutic intervention.

To identify genes involved in creating and maintaining the drug‐tolerant state, we sought to identify transcripts with the greatest differential expression between regressing and drug‐tolerant tumors across all melanoma cell sub‐populations and cancer‐associated fibroblasts. To do this, we conducted bulk mRNA‐seq analysis of BRAF/MEKi‐induced regressing tumors and BRAF/MEKi‐tolerant residual disease. Melanoma cells and fibroblasts were enriched from tumors treated with 3‐day BRAF/MEKi (regressing) and tumors treated with 14‐day BRAF/MEKi (residual disease) using negative selection by depletion of endothelial cells, hematopoietic nucleated cells, red blood cells, and platelets.^[^ ^14^ ^]^ Enrichment was validated by flow cytometry (Figure 1B,C; gating strategy and quantification plot in Figure S1B,C, Supporting Information). Principal component analysis revealed that replicates clustered according to tumor phases (regressing vs residual disease) (Figure 1D). We observed 169 upregulated differentially expressed genes (DEGs) and 21 downregulated DEGs in residual disease compared to regressing tumors (Figure 1E). Along with the heatmap of all DEGs between regressing tumors and residual disease (Figure 1F), these results demonstrate that BRAF/MEKi‐tolerant residual disease acquires a transcriptionally distinct program from regressing tumors, suggesting that this transitional state is governed by unique molecular cues that may underlie persistence and resistance (DEG list in Table S1, Supporting Information).

### 2.2 BRAF/MEKi‐Tolerant Melanomas Have Increased ECM

**Figure 2:**
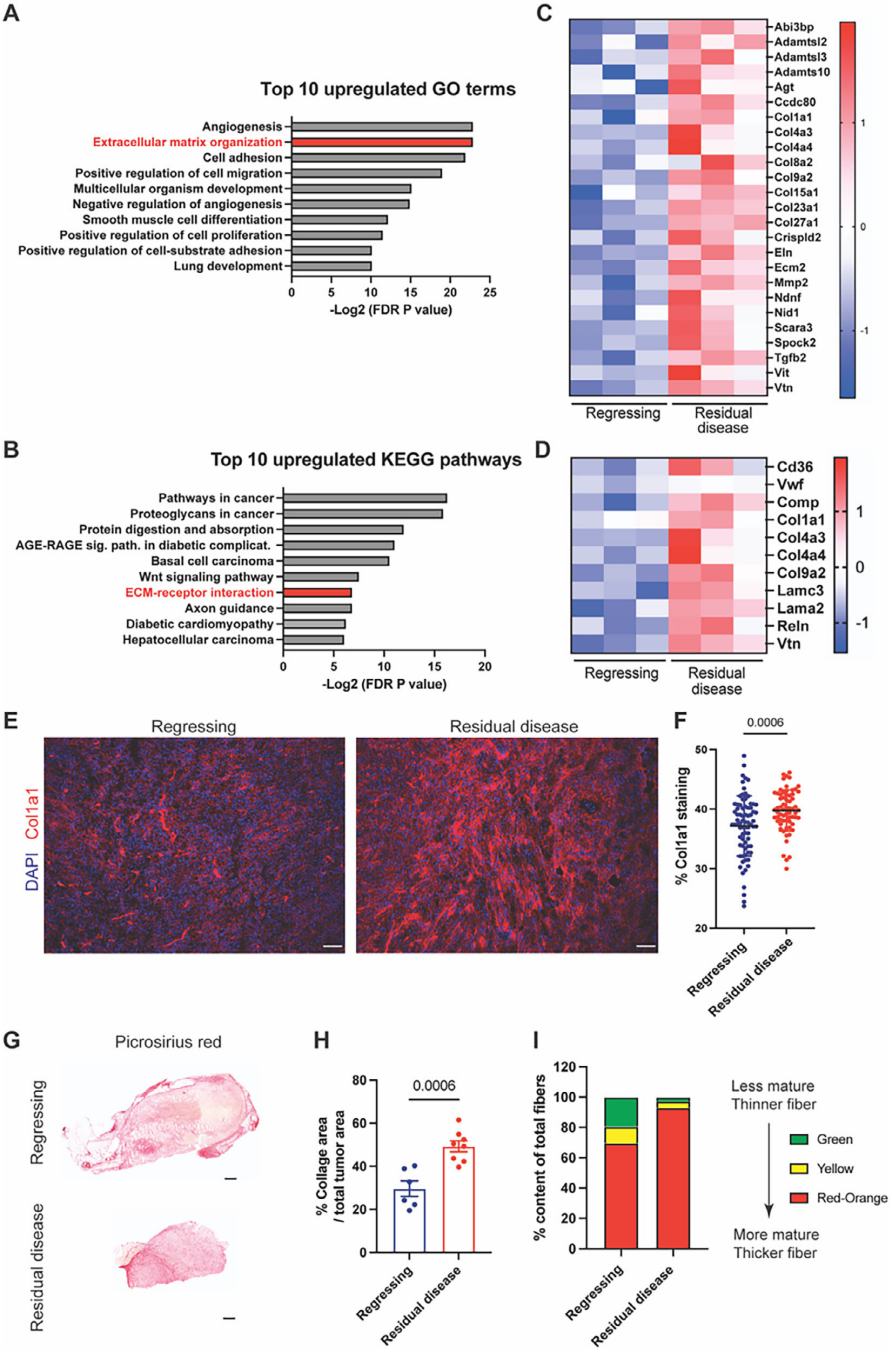
BRAF/MEKi‐tolerant tumors have increased ECM. A) Bar plot showing the top 10 upregulated Gene Ontology (GO) biological processes in residual disease compared to regressing tumors. B) Bar plot showing top 10 upregulated KEGG pathways in residual disease compared to regressing tumors. C) Heatmap showing the differentially expressed genes that are classified in the GO biological process “Extracellular matrix organization” from Figure 2A. D) Heatmap showing the differentially expressed genes classified in the KEGG pathway “ECM‐receptor interaction” from Figure 2B. For both panels in (C) and (D), rows represent genes and columns represent individual tumor samples from regressing or residual disease groups. Gene expression values are scaled by row‐wise Z‐score normalization (mean‐centered across samples for each gene). Red indicates higher expression and blue indicates lower expression relative to the gene's mean expression. E) Representative immunofluorescence images of Col1a1 (red) and nuclear DAPI (blue) of regressing tumors and residual disease treated with BRAF/MEKi. Scale bar, 100 µm. F) Quantification of percent Col1a1 area per field in melanoma tissues. *n* = 6 tumors per group with 77 (regressing tumors) and 65 (residual disease) total microscopy fields analyzed across all tissues in the respective groups. Data presented as mean with SD. Statistics were calculated using a two‐tailed unpaired *t*‐test. G) Representative picrosirius red staining showing collagen deposition, imaged using a ScanScope in regressing tumors and residual disease. Scale bar, 500 µm. H) Quantification of percent collagen area per total tumor area. *n* = 6–8 tumors per group. Data presented as mean with SEM. Statistics were calculated using a two‐tailed unpaired *t*‐test. I) Quantification of collagen maturity and fiber thickness in regressing tumors and residual disease stained with picrosirius red using circular polarized light microscopy. Birefringence hue was quantified as a percent of total fibers. *n* = 2–3 tumors per group, 2–6 fields per tumor section. Data presented as mean.

To identify the biological processes that distinguish the regressing and residual disease states in melanoma, we performed Gene Ontology (GO) and KEGG pathway enrichment analyses on transcripts from both melanoma cells and cancer‐associated fibroblasts (**Figure** **2**A,B). While angiogenesis emerged as the top upregulated GO term, the concurrent enrichment of “negative regulation of angiogenesis” reduced our confidence in angiogenesis as a consistent or actionable feature at this time point. To experimentally interrogate this pathway, we performed immunofluorescence staining for CD31 but observed significantly higher CD31 expression in regressing tumors compared to residual disease, suggesting that angiogenesis may not be sustained or functionally active during the residual phase (Figure S2A,B, Supporting Information).

The Wnt signaling pathway was also enriched in KEGG analysis (Figure 2B). We validated this by immunofluorescence staining of Lef1 and observed significantly elevated expression in residual disease compared to regressing tumors (Figure S2C,D, Supporting Information). However, pharmacologic inhibition of Wnt signaling using WNT‐C59 did not significantly delay the onset of resistance (Figure S2E,F, Supporting Information), arguing against its role as a key driver of therapeutic escape.

After ruling out these candidate pathways, alterations in the extracellular matrix (ECM) emerged as the most consistently enriched category across both GO and KEGG analyses (Figure 2A,B), suggesting a robust shift in ECM‐related processes during the transition to residual disease.

Detailed examination of the transcriptomic data revealed a significant upregulation of ECM‐associated genes in residual disease compared to regressing tumors, indicating dynamic remodeling of the tumor microenvironment (Figure 2C,D). To validate these observations, we performed RT‐qPCR on RNA extracted from enriched melanoma cells and fibroblasts from both regressing and residual tumors. Genes involved in ECM structure and organization, including *Col1a1*, *Col4a3*, *Eln*, *Tgfb2*, and *Lama2*, were consistently upregulated in residual disease (Figure S3A, Supporting Information). Histological analysis further confirmed these transcriptomic changes at the protein level. Immunofluorescence and picrosirius red staining of tumor sections revealed markedly increased collagen deposition in residual disease relative to regressing tumors (Figure 2E–H). Moreover, collagen fiber composition analysis under circularly polarized light microscopy demonstrated a shift toward mature, densely packed collagen fibers, evidenced by an increase in red and orange birefringent fibers and a decrease in immature green fibers in residual tumors (Figure 2I).

To account for the possible confounding effects of tumor size, we compared collagen deposition between vehicle‐treated and fully resistant tumors, both of which had similar volumes. Resistant tumors exhibited significantly more collagen deposition and more mature fiber architecture than vehicle‐treated tumors, supporting the notion that ECM remodeling is a therapy‐induced feature rather than a baseline property of tumor growth (Figure S3B–F, Supporting Information).

To provide a more comprehensive view across treatment phases, we additionally plotted collagen content across all four groups, including vehicle‐treated, regressing, residual disease, and resistant tumors, on a unified graph (Figure S3G, Supporting Information). This analysis revealed a stepwise increase in collagen from vehicle to residual disease, with resistant tumors showing intermediate levels. These findings suggest that ECM remodeling is initiated early during therapy and sustained, though partially attenuated, during the resistant phase.

While prior studies have reported ECM alterations in therapy‐resistant melanomas,^[^ ^15^, ^16^ ^]^ our findings define the post‐regression, residual disease window as the key inflection point at which ECM remodeling initiates. These results suggest that therapy‐induced changes to the ECM are an early and sustained adaptation in the resistant tumor microenvironment, potentially contributing to immune evasion and therapeutic failure.

Having identified early ECM remodeling as a defining feature of therapy‐tolerant residual disease in our preclinical model, we next asked whether similar ECM changes occur during targeted therapy treatment in human melanoma patients.

### 2.3 Collagen and ECM‐Related Genes are Upregulated in Melanoma Patients During and After Targeted Therapy

**Figure 3:**
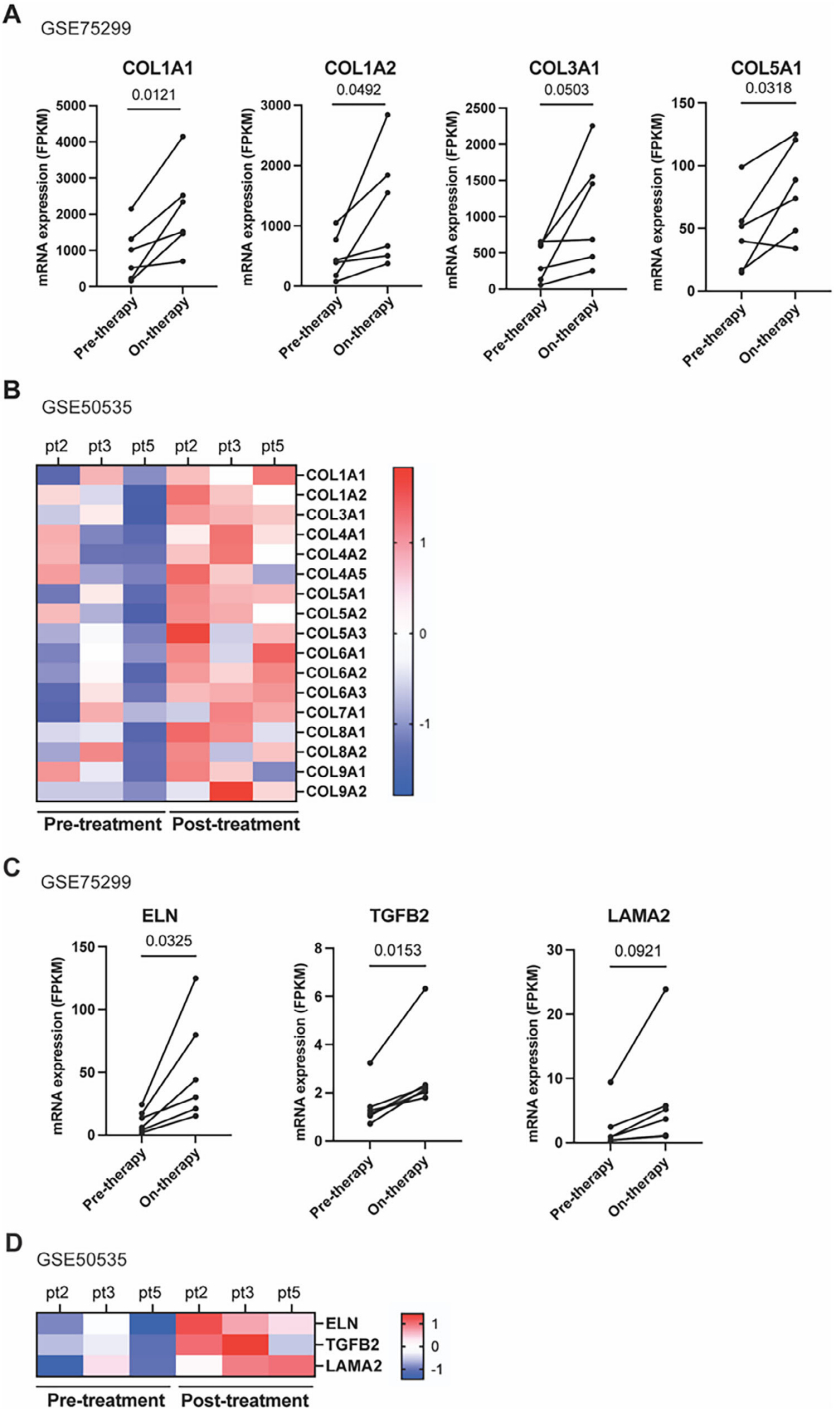
ECM‐related genes are upregulated in on‐ and post‐treatment human patient melanomas over pre‐treatment melanomas. A) Dot plots comparing pre‐therapy and on‐therapy collagen mRNA levels in melanoma patients treated with BRAFi or BRAF/MEKi from GSE75299. Matched patient sample size of *n* = 6. Statistics were calculated using a two‐tailed paired *t*‐test. B) Heatmap showing increased collagen mRNA levels in post‐treatment melanoma patients treated with BRAF/MEKi over pre‐treatment melanoma patients from GSE50535. Matched patient sample size of *n* = 3. Gene expression values were normalized by row‐wise Z‐score (mean‐centered per gene across samples). Red indicates higher expression and blue indicates lower expression relative to the gene's average. C) Dot plots comparing pre‐therapy and on‐therapy ECM‐related mRNA levels in melanoma patients treated with BRAFi or BRAF/MEKi from GSE75299. Matched patient sample size of *n* = 6. Statistics were calculated using a two‐tailed paired *t*‐test. D) Heatmap showing increased ECM‐related mRNA levels in post‐treatment melanoma patients treated with BRAF/MEKi over pre‐treatment melanoma patients from GSE50535. Matched patient sample size of *n* = 3. Gene expression values were normalized by row‐wise Z‐score (mean‐centered per gene across samples). Red indicates higher expression and blue indicates lower expression relative to the gene's average.

To evaluate the clinical relevance of our preclinical findings, we analyzed two publicly available gene expression datasets (GSE75299 and GSE50535) comprising matched tumor biopsies from melanoma patients collected before, during, and after treatment with BRAF inhibitors (BRAFi) or combined BRAF/MEK inhibitors (BRAF/MEKi) (Table S2, Supporting Information).^[^ ^17^, ^18^ ^]^ These datasets provided an opportunity to evaluate dynamic changes in ECM‐related gene expression in response to targeted therapy. In the GSE75299 cohort, collagen‐related genes including *COL1A1*, *COL1A2*, *COL3A1*, and *COL5A1* were significantly upregulated in on‐treatment samples compared to pre‐treatment biopsies from the same patients (**Figure** **3**A). A similar increase in collagen gene expression was observed in GSE50535 when comparing matched pre‐ and post‐treatment samples (Figure 3B). Beyond collagen, we also observed consistent upregulation of additional ECM‐related genes, such as *ELN*, *TGFB2*, and *LAMA2*, in both datasets, with elevated expression during and after therapy compared to pre‐treatment levels (Figure 3C,D). These findings mirror the transcriptional shifts identified in our mouse models of residual disease and therapy resistance. Collectively, these patient data validate that ECM remodeling, particularly increased collagen expression, is a conserved feature of targeted therapy response in melanoma. This supports the translational relevance of targeting the ECM during residual disease as a strategy to extend the efficacy of BRAF/MEKi treatment.

### 2.4 Targeting the ECM Delays the Onset of Resistance to BRAF/MEKi

**Figure 4:**
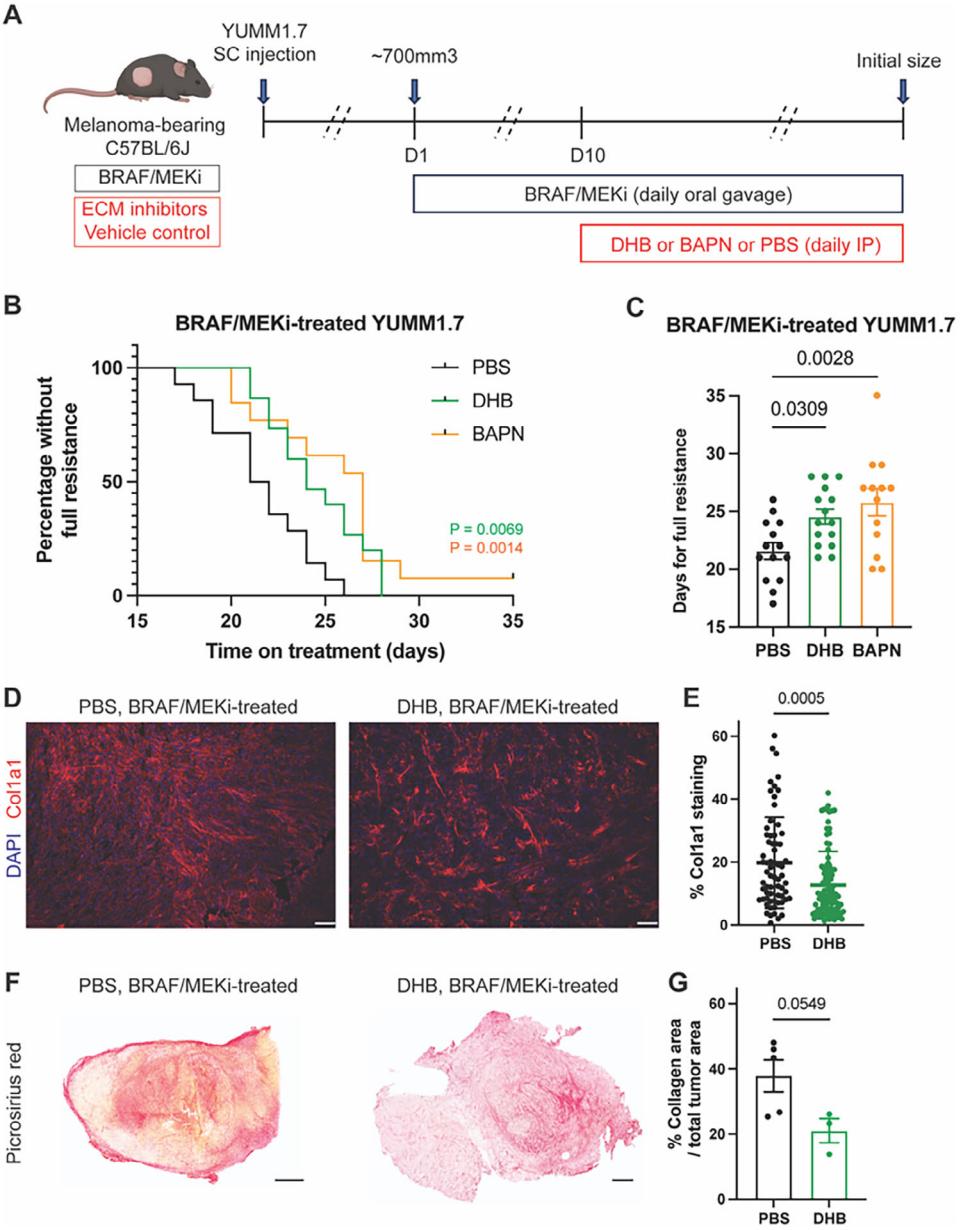
Targeting the ECM delays the onset of resistance to BRAF/MEKi. A) Scheme illustrating targeting ECM in YUMM1.7‐bearing mice. BRAF/MEKi was administered daily by oral gavage starting when tumors reached ≈700mm^3^ in size. DHB, BAPN, or PBS (vehicle control) was injected intraperitoneally daily from day 10. Tumors were collected when reaching the initial size before BRAF/MEKi treatment. B) Kaplan–Meier curve for BRAF/MEKi‐treated mice bearing YUMM1.7 receiving PBS, DHB, or BAPN. *n* = 14, 15, and 13 tumors in the PBS, DHB, and BAPN groups, respectively. The “percentage without full resistance” refers to the proportion of tumors in each cohort that have not yet reached full resistance. Statistics were calculated using the Log‐rank (Mantel‐Cox) test. C) Days for BRAF/MEKi‐treated mice with PBS, DHB, or BAPN injection to develop full resistance (tumors reached the initial size before BRAF/MEKi treatment, i.e., ≈700mm^3^ in size). *n* = 14, 15, and 13 tumors in the PBS, DHB, and BAPN groups, respectively. Data presented as mean with SEM. Statistics were calculated using one‐way ANOVA with Dunnett's multiple comparisons test. D) Representative immunofluorescence images of Col1a1 (red) and nuclear DAPI (blue) of BRAF/MEKi‐treated resistant tumors in the indicated treatment groups. Scale bar, 100 µm. E) Quantification of percent Col1a1 area per field in melanoma tissues. *n* = 4 tumors per group, with 66 (PBS) and 93 (DHB) total microscopy fields analyzed across all tissues in respective groups. Data presented as mean with SD. Statistics were calculated using a two‐tailed unpaired *t*‐test. F) Representative picrosirius red stains of BRAF/MEKi‐treated resistant tumors imaged using a ScanScope in the indicated treatment groups. Scale bar, 500 µm. G) Quantification of percent collagen area per total tumor area. *n* = 3–5 tumors per group. Data presented as mean with SEM. Statistics were calculated using a two‐tailed unpaired *t*‐test. Figure 4A was created with BioRender.com.

Having identified a pronounced increase in ECM remodeling during the residual disease phase, we next tested whether therapeutically targeting the ECM at this critical window could delay the emergence of resistance. To disrupt collagen architecture, we employed two pharmacological inhibitors: 3,4‐dihydroxybenzoic acid (DHB), which inhibits collagen prolyl 4‐hydroxylase and thereby prevents collagen triple helix formation, and β‐aminopropionitrile (BAPN), a lysyl oxidase inhibitor that blocks collagen crosslinking.^[^ ^19^, ^20^, ^21^, ^22^ ^]^ Using our established melanoma model, we initiated treatment with DHB or BAPN on day 10 of BRAF/MEKi therapy, coinciding with the onset of residual disease, while maintaining continuous BRAF/MEKi administration (**Figure** **4**A). Both ECM‐targeting agents significantly delayed tumor regrowth compared to BRAF/MEKi alone, as defined by the time required for tumors to return to their pre‐treatment volume (≈700 mm^3^) (Figure 4B,C; Figure S4A, Supporting Information). These results indicate that modulating ECM organization during residual disease can enhance the durability of BRAF/MEKi and suppress the progression to full resistance. Consistent with previous studies in other models,^[^ ^19^, ^23^ ^]^ we confirmed that enzymatic inhibition by DHB or BAPN reduced collagen deposition or impaired collagen maturation, respectively, in YUMM1.7 tumors (Figure 4D–G; Figure S4B–D, Supporting Information).

### 2.5 High‐Density Collagen Physically Restricts CD8⁺ T Cell Accessibility in Residual Disease

**Figure 5:**
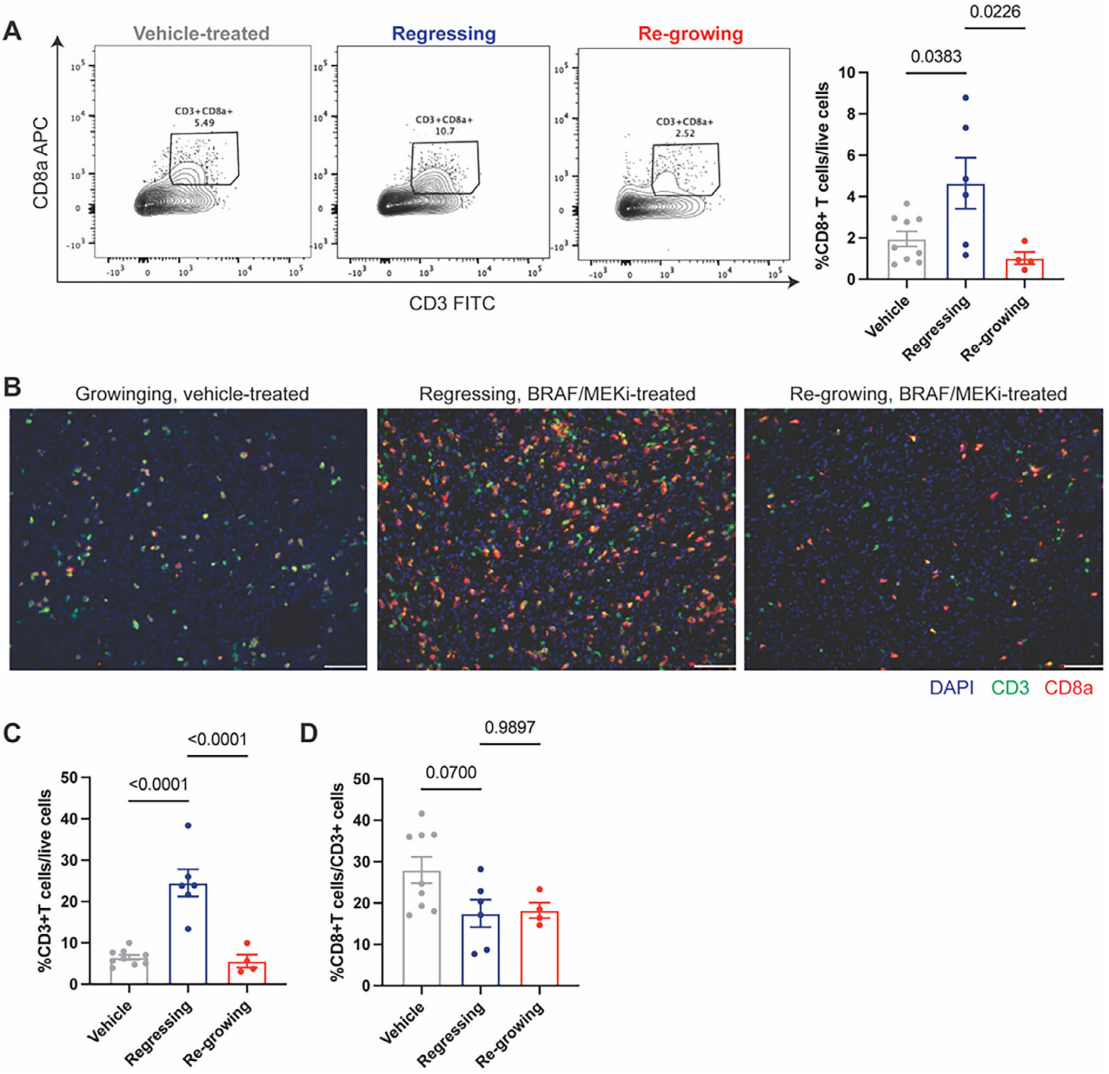
On‐therapy regrowing tumors have significantly fewer CD8+ T cells compared to on‐therapy regressing tumors. A) Flow cytometry analysis of tumor‐infiltrating CD8+ T cells gated on live/CD45+ cells in vehicle control‐treated, regressing, and regrowing tumors treated with BRAF/MEKi. Left, representative flow cytometry plots. Numbers on the plots represent the percentage of cells within each gate. Right, quantification of tumor‐infiltrating CD8+ T cells in each group. *n* = 9, 6, and 4 tumors in the vehicle‐treated group, regressing group, and regrowing group, respectively. Data presented as mean with SEM. Statistics were calculated using ordinary one‐way ANOVA. B) Representative immunofluorescence images of CD3 (green), CD8a (red), and nuclear DAPI (blue) of vehicle‐treated, regressing, and regrowing tumors treated with BRAF/MEKi. Scale bar, 100 µm. C) Quantification of tumor‐infiltrating CD3⁺ T cells as a percentage of live cells. D) Quantification of CD8⁺ T cells as a percentage of total CD3⁺ T cells. Vehicle‐treated (*n* = 9), regressing (*n* = 6), and re‐growing (*n* = 4) tumors. Data presented as mean with SEM. Statistics were calculated using ordinary one‐way ANOVA.

Next, we sought to explore the mechanisms underlying the delayed onset of therapy resistance resulting from inhibition of collagen fiber development. We observed that CD8⁺ T cells were relatively scarce in BRAF/MEKi‐insensitive, on‐therapy regrowing melanoma tumors, but abundant in BRAF/MEKi‐sensitive, on‐therapy regressing tumors, as quantified in vehicle‐treated, regressing, and regrowing tumors (**Figure** **5**A,B). These findings are consistent with the notion that therapy‐induced ECM remodeling may structurally restrict immune cell access during disease progression.

To determine whether the observed CD8⁺ T cell dynamics reflected a selective change or a broader shift in lymphocyte infiltration, we quantified CD3⁺ T cells. CD3⁺ T cell abundance mirrored CD8⁺ T cell trends across treatment stages, with significantly higher infiltration during tumor regression (Figure 5C). However, the proportion of CD8⁺ T cells among CD3⁺ cells remained consistent across groups (Figure 5D), indicating that the decline in CD8⁺ T cell numbers during resistance reflects a general reduction in T cell infiltration rather than selective CD8⁺ T cell loss.

**Figure 6:**
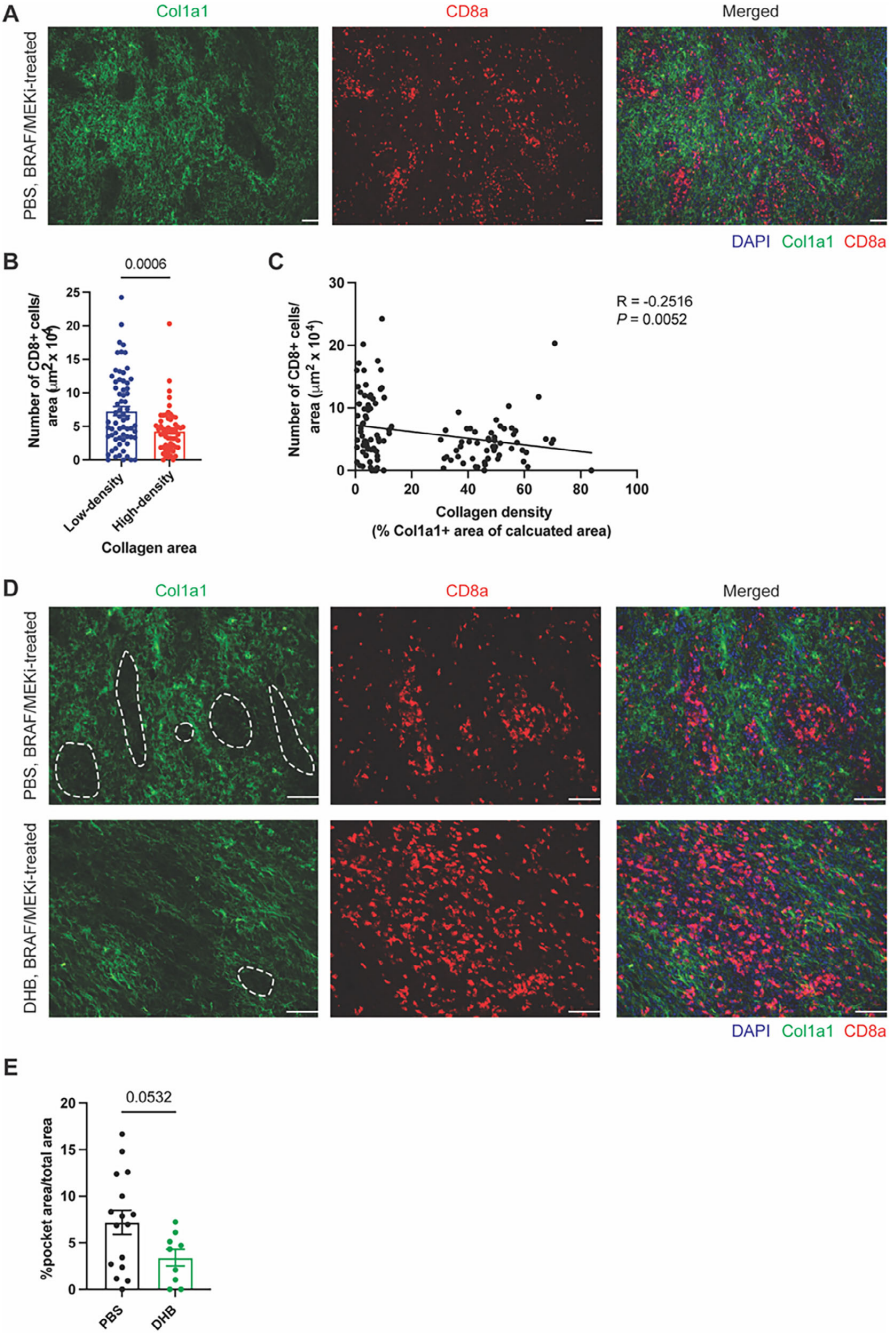
High‐density collagen restricts CD8+ T cell accessibility. A) Representative immunofluorescence images of Col1a1 (green), CD8a (red), and nuclear DAPI (blue) in BRAF/MEKi‐treated residual disease. Scale bar, 100 µm. B) Quantification of tumor‐infiltrating CD8+ T cells in low‐ and high‐density collagen areas in residual disease. *n* = 4 tumors with 66 (low‐density) and 56 (high‐density) collagen areas analyzed. Low‐ and high‐density collagen areas were defined as % Col1a1+ area <15% and >30%, respectively. Data presented as mean with SEM. Statistics calculated using a two‐tailed unpaired *t*‐test. C) Scatter plot showing the negative correlation between the collagen density and the number of CD8+ cells in residual disease. The Pearson correlation (R) between the plotted values and the two‐sided *p* values is shown in the upper right corner. D) Representative immunofluorescence images of Col1a1 (green), CD8a (red), and nuclear DAPI (blue) in BRAF/MEKi‐treated residual disease in the indicated treatment group. Scale bar, 100 µm. A representative “pocket” area (defined as <5% Col1a1⁺ signal and localized morphology) is outlined with a dashed line in each Col1a1 image. These regions were identified and quantified using Fiji in a blinded manner, as described in the Methods. E) Quantification of % pocket areas in BRAF/MEKi‐treated residual disease in the indicated treatment group. *n* = 4 (PBS) and 3 (DHB) tumors with 16 (PBS) and 9 (DHB) total microscopy fields analyzed in respective groups. Data presented as mean with SEM. Statistics calculated using a two‐tailed unpaired *t*‐test.

To investigate whether collagen density spatially correlates with CD8⁺ T cell localization, we performed co‐immunofluorescence staining for Col1a1 and CD8a in therapy‐tolerant residual disease. Notably, CD8⁺ T cells were significantly enriched in low‐density collagen regions, while high‐density collagen areas were largely devoid of CD8⁺ T cells (**Figure** **6**A–C). This distinct distribution pattern supports the hypothesis that a dense collagen matrix restricts immune cell infiltration, forming spatial “barriers” within the tumor microenvironment. Further, immunofluorescence staining showed that CD8⁺ T cells in residual tumors tend to accumulate in discrete collagen‐low “pockets” rather than being evenly dispersed (Figure 6D,E). Importantly, treatment with the ECM‐modulating agent DHB, which reduces collagen deposition (Figure 4D–G; Figure S4B,C, Supporting Information), did not expand the area of these pockets but instead facilitated a more uniform distribution of CD8⁺ T cells throughout the tumor tissue. This observation suggests that DHB diminishes collagen‐mediated immune exclusion, thereby improving CD8⁺ T cell access to tumor cells in residual disease. Together, these results demonstrate that dense collagen architecture physically restricts CD8⁺ T cell infiltration during the residual disease phase. These findings support a model in which collagen organization contributes to immune exclusion by forming physical barriers, potentially limiting CD8⁺ T cell‐mediated tumor control.

### 2.6 CD8+ T Cells are Required for ECM‐Mediated Delay of BRAF/MEKi Resistance

**Figure 7:**
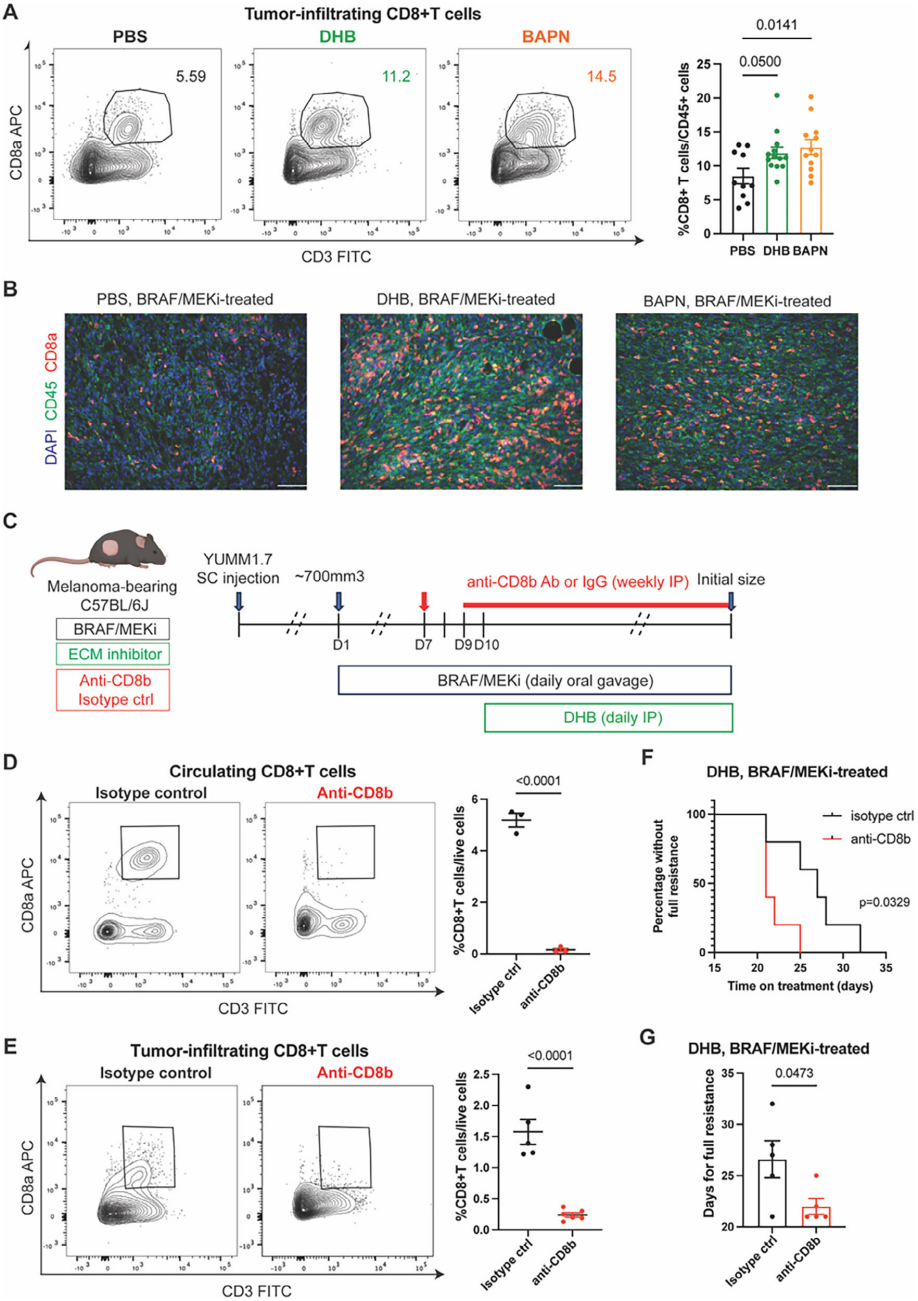
Targeting ECM deposition enhances anti‐tumor immunity. A) Flow cytometry analysis of tumor‐infiltrating CD8+ T cells gated on live/CD45+ cells in BRAF/MEKi‐treated mice injected intraperitoneally with PBS, DHB, or BAPN. Left, representative flow cytometry plots. Numbers on the plots represent the percentage of cells within each gate. Right, quantification of tumor‐infiltrating CD8+ T cells in each group. *n* = 10, 13, and 12 tumors in the PBS, DHB, and BAPN groups, respectively. Data presented as mean with SEM. Statistics calculated using one‐way ANOVA with Dunnett's multiple comparisons test. B) Representative immunofluorescence images of CD45 (green), CD8a (red), and nuclear DAPI (blue) in the indicated BRAF/MEKi‐treated tumor groups. Scale bar, 100 µm. C) Scheme illustrating CD8+ T cell depletion in YUMM1.7‐bearing mice treated with BRAF/MEKi + DHB. BRAF/MEKi were administered by daily oral gavage when tumors reached ≈700mm^3^ in size. DHB was injected intraperitoneally daily from day 10. CD8+ T cells were depleted by IP injection of anti‐CD8b antibodies. An IgG1 isotype control antibody was used in control animals. Tumors were collected when reaching the initial volume prior to BRAF/MEKi treatment. D) Flow cytometry analysis of circulating CD8+ T cells gated on live/CD45+ cells in BRAF/MEKi + DHB‐treated mice injected intraperitoneally with anti‐CD8b antibodies or isotype control. Left, representative flow cytometry plots. Numbers on the plots represent the percentage of cells within each gate. Right, quantification of circulating CD8+ T cells. *n* = 3 mice in both groups. Data presented as mean with SEM. Statistics were calculated using a two‐tailed unpaired *t*‐test. E) Flow cytometry analysis of tumor‐infiltrating CD8+ T cells gated on live/CD45+ cells in BRAF/MEKi + DHB‐treated mice injected intraperitoneally with anti‐CD8b antibodies or isotype control. Left, representative flow cytometry plots. Numbers on the plots represent the percentage of cells within each gate. Right, quantification of tumor‐infiltrating CD8+ T cells. *n* = 5 and 6 tumors in anti‐CD8b and isotype control respectively. Data presented as mean with SEM. Statistics were calculated using a two‐tailed unpaired *t*‐test. F) Kaplan–Meier curve for BRAF/MEKi + DHB‐treated mice bearing YUMM1.7 receiving anti‐CD8b antibodies or isotype control. The “percentage without full resistance” refers to the proportion of tumors in each cohort that have not yet reached full resistance. Statistics were calculated using the Log‐rank (Mantel‐Cox) test. G) Days for BRAF/MEKi + DHB‐treated mice with anti‐CD8b antibodies or isotype control injection to develop full resistance (tumors reached the initial size before BRAF/MEKi treatment, i.e., ≈700mm^3^ in size). *n* = 5 tumors in both groups. Data presented as mean with SEM. Statistics were calculated using a two‐tailed unpaired *t*‐test. Figure 7C was created with BioRender.com.

Given that high‐density collagen restricted CD8⁺ T cell access to residual disease, we hypothesized that inhibiting collagen deposition may delay therapy resistance by enhancing CD8⁺ T cell‐mediated tumor clearance. To test this, we quantified intratumoral CD8⁺ T cell infiltration following ECM modulation. Flow cytometry analysis revealed that both DHB and BAPN significantly increased CD8⁺ T cell infiltration compared to vehicle‐treated tumors, without affecting the overall abundance of CD45⁺ leukocytes (**Figure** **7**A,B; Figure S5A; gating strategy in Figure S5B, Supporting Information). Moreover, the proportion of CD3⁺ T cells among total CD45⁺ immune cells remained unchanged across treatment groups (Figure S5C, Supporting Information), suggesting that ECM‐targeting therapies selectively enhance CD8⁺ T cell access rather than broadly altering immune cell recruitment.

To determine whether these effects were tumor‐specific, we also analyzed circulating CD8^+^ T cells in the blood and found no significant changes in CD8⁺ T cell frequency following DHB or BAPN treatment (Figure S5D, Supporting Information), indicating a localized effect within the tumor microenvironment. Finally, we evaluated whether ECM inhibition modulates CD8⁺ T cell exhaustion and observed modest increases in TIGIT⁺ and NKG2A⁺ CD8⁺ T cells in DHB‐ and BAPN‐treated tumors, while PD‐1 expression remained unchanged (Figure S5E–G, Supporting Information). These findings suggest that ECM‐targeting therapies enhance local CD8⁺ T cell infiltration and partially modulate their functional state, without inducing systemic immune alterations.

Since a previous study reported that NK cells are embedded in the collagen‐rich ECM surrounding the tumor foci in beta‐2 microgobulin^−/−^ melanoma,^[^ ^20^ ^]^ we also quantified tumor‐infiltrating NK cells in DHB‐, BAPN‐, and vehicle‐treated melanomas. However, no significant differences in NK cell frequencies were observed in DHB‐ or BAPN‐treated tumors compared to controls (Figure S6A; gating strategy in S6B, Supporting Information), indicating a selective effect on CD8⁺ T cells.

To determine whether the delay in resistance mediated by ECM inhibition was CD8⁺ T cell–dependent, we treated YUMM1.7 tumor‐bearing mice with DHB and concurrently depleted CD8⁺ T cells using an anti‐CD8b antibody (Figure 7C). Depletion was confirmed by flow cytometry in both blood and tumors (Figure 7D,E). As expected, DHB no longer delayed resistance onset in the absence of CD8⁺ T cells, and tumors progressed to full resistance with kinetics similar to untreated controls (Figure 7F,G). These findings demonstrate that the therapeutic benefit of ECM inhibition during residual disease relies on CD8⁺ T cell activity. By enhancing T cell accessibility to tumor cells, ECM‐targeting agents promote immune‐mediated suppression of resistant clones, thereby extending the efficacy window of BRAF/MEKi treatment.

To further assess whether NK cells contribute functionally to the therapeutic benefit of ECM inhibition, we depleted NK cells using anti‐ASGM1 antibodies in DHB‐treated, BRAF/MEKi‐co‐administered mice. NK cell depletion did not significantly alter resistance kinetics or the time to full tumor regrowth (Figure S6C,D, Supporting Information), suggesting that NK cells are not required for the delay in resistance observed with ECM‐targeting therapy.

Together, these results indicate that therapy‐induced collagen accumulation broadly restricts T cell infiltration during the emergence of resistance. ECM‐targeting agents selectively alleviate this barrier to restore local CD8⁺ T cell access without altering overall leukocyte recruitment or systemic T cell distribution. These findings reconcile the decline in tumor‐infiltrating T cells observed in tumors developing resistance with the CD8⁺‐dependent therapeutic benefit of ECM inhibition, highlighting the ECM as a critical regulator of immune accessibility in melanoma.

Having established that collagen restricts CD8⁺ T cell function in our preclinical model, we next investigated whether this relationship is also evident in human melanoma.

### 2.7 Collagen Expression Negatively Correlates with CD8^+^ T Cell Infiltration and Survival in Melanoma Patients

**Figure 8:**
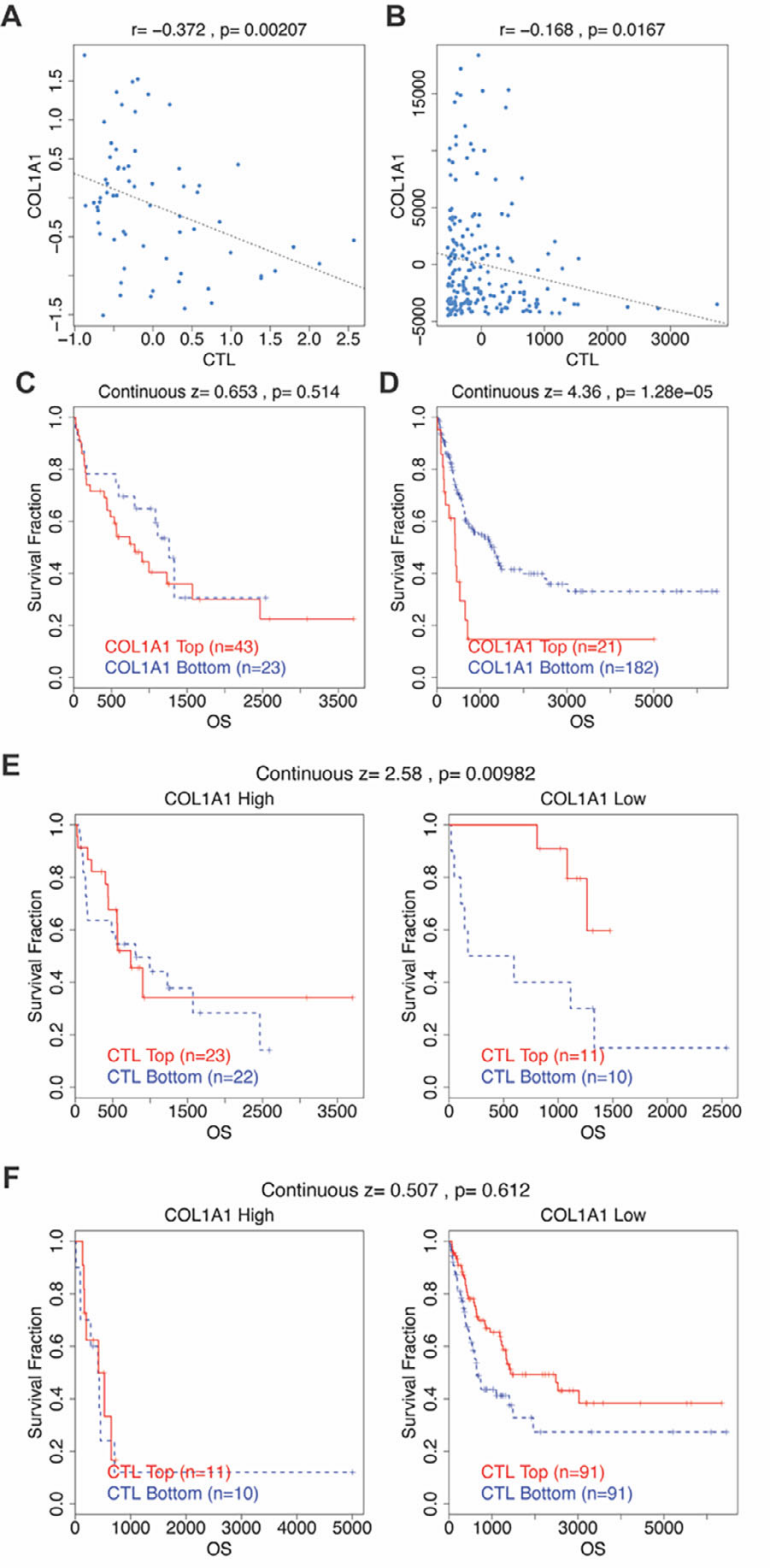
Collagen negatively correlates with CD8+ T cell infiltration and survival in melanoma patients. A,B) Scatter plots showing the negative correlation between *COL1A1* gene expression and cytotoxic T lymphocyte (CTL) infiltration level in the GSE8401 primary and metastatic melanoma datasets (*n* = 66, A) or the GSE65904 metastatic melanoma dataset (*n* = 203, B). Each blue dot represents one patient sample. The *y‐*axis represents gene expression values as originally reported in the respective datasets. C,D) Kaplan–Meier plots of overall survival (OS, in days) for melanoma patients with low (Bottom, shown in blue) and high (Top, shown in red) *COL1A1* expression levels in the GSE8401 primary and metastatic melanoma dataset (C) or the GSE65904 metastatic melanoma dataset (D). E,F) Kaplan–Meier plots of overall survival (OS, in days) stratified by CTL levels within *COL1A1*‐High (left panels) and *COL1A1*‐Low (right panels) tumors in the GSE8401 (E) and GSE65904 (F) melanoma datasets. Samples were split by *COL1A1* expression to assess whether CTL levels are differentially prognostic. CTL Top (high CTL, red) and CTL Bottom (low CTL, blue) groups are shown. The *p*‐values indicate interaction effects as computed using the TIDE framework, evaluating whether the prognostic impact of CTL differs by *COL1A1* expression. The analyses presented in this figure were derived from the computational framework TIDE, developed by Jiang et al.^[^ ^24^ ^]^

To further explore the clinical significance of collagen‐mediated immune exclusion, we assessed the relationship between *COL1A1* expression, CD8⁺ T cell infiltration, and patient survival in melanoma. Due to the limited sample sizes in publicly available datasets of melanoma patients treated with BRAF/MEKi, we turned to broader transcriptomic datasets of primary and metastatic melanoma using TIDE (Tumor Immune Dysfunction and Exclusion), a computational framework developed to infer immune cell infiltration and dysfunction from gene expression profiles.^[^ ^24^ ^]^ TIDE defines cytotoxic lymphocytes (CTLs) primarily as CD8⁺ T cells. In both the GSE8401 dataset (primary and metastatic melanoma)^[^ ^25^ ^]^ and the GSE65904 dataset (metastatic melanoma),^[^ ^26^ ^]^ *COL1A1* expression was significantly negatively correlated with CTL infiltration, suggesting that higher collagen levels are associated with reduced CD8⁺ T cell presence in human melanomas (**Figure** **8**A,B). These findings are consistent with our spatial imaging and functional data from the mouse model, in which dense collagen limited CD8⁺ T cell accessibility to tumors.

We next examined the relationship between *COL1A1* expression and patient survival. In GSE65904, patients with low *COL1A1* expression (“COL1A1 Bottom”) exhibited significantly improved overall survival compared to those with higher expression (Figure 8D). A similar trend was observed in GSE8401, though it did not reach statistical significance, likely due to smaller sample size or cohort heterogeneity (Figure 8C).

To assess whether collagen levels modulate the prognostic impact of CTL infiltration, we stratified patients by both *COL1A1* and CTL expression. In GSE8401, we observed a significant interaction between COL1A1 and CTL levels: high CTL infiltration (“CTL Top”) was associated with better survival only in COL1A1‐low tumors (Figure 8E). While this interaction was not statistically significant in GSE65904 (Figure 8F), a similar directional trend was observed, with high CTL levels modestly associated with improved survival only in COL1A1‐low tumors.

Although the strength of association varied across datasets, the overall pattern aligns with our preclinical findings that collagen‐rich ECM diminishes the immune‐protective effect of CD8⁺ T cell infiltration. These clinical transcriptomic data, while not perfectly matched to our preclinical models, reinforce the translational relevance of our findings and support targeting collagen remodeling as a strategy to improve immunosurveillance and patient outcomes in melanoma.

## 3 Discussion

The ECM, though classically viewed as a structural scaffold, actively shapes cell signaling, immune infiltration, and drug response.^[^ ^27^ ^]^ Previous studies in melanoma have shown that collagen accumulation in BRAF‐inhibited xenografts promotes resistance through reactivation of MAPK signaling or survival pathways such as DDR1/2 and integrin β1/FAK.^[^ ^15^, ^16^, ^28^, ^29^, ^30^, ^31^ ^]^ In those studies, the outcome of changes to the ECM was examined primarily through the lens of melanoma cell–intrinsic adaptations. Our work differs in two key ways: 1) we define the temporal window during which ECM remodeling first emerges, corresponding with residual disease, and 2) we reveal that ECM remodeling contributes to resistance by physically excluding CD8⁺ T cells, thereby impairing immune surveillance.

Most preclinical studies investigating BRAF/MEKi resistance, particularly those exploring ECM alterations as a mechanism, have relied on immune‐deficient models, which cannot capture the complex interplay between tumor cells, the extracellular matrix, and host immunity.^[^ ^15^, ^16^, ^30^ ^]^ Here, using an immune‐competent mouse model of BRAF‐mutant melanoma, we defined dynamic changes in ECM gene expression across two critical treatment phases: active regression and therapy‐tolerant residual disease. Bulk RNA sequencing of enriched melanoma cells and fibroblasts revealed a marked induction of ECM‐related genes, particularly collagen, during the residual disease phase. Histological analyses corroborated these findings, demonstrating increased collagen deposition and remodeling within tumors during this window. Importantly, we observed similar ECM signatures in human melanoma datasets from patients treated with BRAFi or BRAF/MEKi, suggesting that therapy‐induced ECM alterations are a conserved and clinically relevant response. These findings highlight a previously underappreciated stromal adaptation that emerges during therapy and may contribute to immune exclusion and the eventual development of drug resistance.

By selectively inhibiting collagen deposition or crosslinking during residual disease using DHB or BAPN, we significantly delayed tumor relapse in a CD8⁺ T cell‐dependent manner. CD8⁺ T cell depletion abrogated this therapeutic benefit, confirming that immune exclusion, not only tumor‐intrinsic survival, mediates ECM‐associated resistance. This finding, linking therapy‐induced ECM remodeling to impaired T cell access and functional resistance, adds a new dimension to our understanding of the TME in melanoma. While prior studies have noted that dense collagen matrices can form physical barriers to T cells in other cancers,^[^ ^32^, ^33^ ^]^ our study uniquely demonstrates that ECM remodeling actively contributes to immune exclusion specifically during the residual disease phase of melanoma therapy, representing a previously unrecognized driver of resistance in this context.

Spatial mapping further revealed that CD8⁺ T cells preferentially accumulate in collagen‐low “pockets,” avoiding high‐density collagen regions within residual disease. This pattern was reversed by DHB treatment, which decreased overall collagen content and enabled more uniform T cell distribution. In line with this, patient tumor datasets revealed an inverse correlation between COL1A1 expression and CD8⁺ T cell infiltration, and the survival benefit associated with high CD8⁺ T cell levels was only observed in tumors with low collagen expression.

Interestingly, although NK cells have been shown to respond to collagen‐modulating therapies in MHC‐I–deficient B16‐F10 models,^[^ ^20^ ^]^ we did not observe increased NK cell recruitment following ECM inhibition in our MHC‐I–expressing melanoma model. This discrepancy may reflect differences in tumor type, immune contexture, and treatment timing (on‐therapy residual disease vs off‐therapy growing phase).

One limitation of our study is that RNA‐seq was not performed on fully resistant or vehicle‐treated tumors. However, histological analyses revealed that collagen deposition was significantly higher in resistant tumors compared to vehicle‐treated controls, despite similar tumor sizes. Combined with the upregulation of ECM‐related genes observed during the residual disease phase, these findings suggest that ECM remodeling is driven by therapy exposure rather than tumor burden or inherent growth characteristics. While our focus was on collagen as the most abundant ECM component, our transcriptomic analyses also identified other matrix elements, including TGFB2, elastin, and laminin, as upregulated during residual disease. These components may similarly contribute to immune evasion and resistance, and future studies should explore their functional roles and therapeutic tractability. A further limitation is the current scarcity of patient datasets with paired samples before, during, and after BRAF/MEKi treatment, limiting comprehensive clinical validation of ECM–immune interactions. Expanding these resources will be critical for understanding why some ECM‐targeting strategies fail and how they might be optimized. Although ECM‐targeting strategies such as DHB and BAPN delay resistance onset, tumors ultimately recur in most mice. This suggests that additional mechanisms, such as MAPK pathway reactivation, metabolic rewiring, or immune suppression, may drive late resistance, and should be investigated in future studies.

In summary, our study uncovers a previously unrecognized stromal mechanism of resistance in melanoma: collagen‐rich ECM remodeling during residual disease physically excludes CD8⁺ T cells, enabling tumor persistence and subsequent relapse. By targeting ECM remodeling during this transient therapeutic window, we restore CD8⁺ T cell access and delay resistance in a CD8⁺ T cell‐dependent fashion. This work positions residual disease as a tractable immunosuppressive niche and highlights ECM remodeling as a dynamic, targetable feature of the tumor microenvironment. Beyond its role as a structural scaffold or mediator of survival signaling, we reveal that the ECM actively regulates immune accessibility during therapy. Given the widespread presence of stromal barriers and immune exclusion across solid tumors, these findings may inform generalizable strategies that integrate stromal modulation with molecular‐ or immune‐based therapies to improve durable responses.

## 4 Experimental Section

### Cell Lines

YUMM1.7 melanoma cells (ATCC, RRID: CVCL_JK16) were cultured in DMEM/F‐12 supplemented with 10% FBS, 1% non‐essential amino acids, and penicillin/streptomycin at 37 °C in 5% CO_2_. Cells were confirmed to be mycoplasma‐negative, and no contamination was detected during the course of the study.

### Mouse Models

C57BL/6J mice were purchased from The Jackson Laboratory and bred in‐house. For syngeneic immunocompetent melanoma mouse models, age‐matched 7‐ to 12‐week‐old male mice were injected with YUMM1.7 (1 × 10^6^) subcutaneously into right and left flanks. Tumor volume was measured daily using a digital caliper (V = W^2^ x L/2). Mice received BRAF/MEKi therapy with a dose of 25 mg kg^−1^ dabrafenib (Medchem Express, catalog no. HY‐14660) and 0.15 mg kg^−1^ trametinib (Medchem Express, catalog no. HY‐10999). Dabrafenib and trametinib were initially dissolved in DMSO and subsequently diluted into carboxymethylcellulose (CMC; 0.5% w/v), Tween 80 (0.05% v/v) in sterile PBS to a final volume of 200 µL and administered to mice daily by oral gavage. Mice bearing YUMM1.7 melanomas were treated with BRAF/MEKi when tumors reached ≈700mm^3^. Full resistance was defined as the time point at which an individual tumor, under continuous BRAF/MEKi treatment, resumes progressive growth and reaches a volume equal to or greater than its pretreatment baseline size (≈700 mm^3^).

### Melanoma Cell and Fibroblast Enrichment

To enrich melanoma cells and cancer‐associated fibroblasts, the negative selection approach reported by Slipicevic et al. was performed.^[^ ^14^ ^]^ Tumors were collected and dissociated in DMEM/F12 media containing collagenase Type I (20 mg mL^−1^) (Worthington, catalog no. LS004196) at 37 °C for 75 min. Digested materials were filtered through 70 µm cell strainers to obtain a single‐cell suspension. An anti‐CD16/32 Fc receptor blocker (Biolegend, catalog no. 156603) was added to the cell suspension. Melanoma cells and cancer‐associated fibroblasts were enriched by negative selection through depletion of endothelial cells, hematopoietic nucleated cells, red blood cells and platelets using a cocktail of biotinylated antibodies including anti‐CD31 (clone 390, Biolegend, catalog no. 102404), anti‐CD45 (clone 30‐F11, Biolegend, catalog no. 103103), anti‐TER‐119 (clone TER‐119, Biolegend, catalog no. 116203), and anti‐CD41 (clone MWReg30, Biolegend, catalog no. 133930) antibodies followed by Dynabeads Biotin Binder (Invitrogen, catalog no. 11047) according to the instruction manual. Unbound cells were collected and underwent downstream experiments.

### RNA Isolation and RT‐qPCR

Before RNA extraction, tumors were dissociated and subjected to negative selection as described above. This enrichment strategy ensured the resulting RNA predominantly reflected melanoma cells and cancer‐associated fibroblasts, minimizing confounding effects from immune and vascular compartments. The extraction of total RNA from enriched melanoma cells and cancer‐associated fibroblasts was performed with the RNeasy Plus Micro Kit (Qiagen, catalog no. 74034) according to the manufacturer's protocol. RNA was transcribed into cDNA using the High‐Capacity RNA‐to‐cDNA kit (Applied Biosystems, catalog no. 4387406). cDNA amplification was conducted using the Luna Universal qPCR Master Mix (New England Biolabs, catalog no. M3003) according to the manufacturer's instructions. The primer sequences utilized in the study are listed in Table S3 (Supporting Information).

### Bulk mRNA Sequencing and Analysis

To compare gene expression between regressing and residual disease tumors while minimizing batch effects, tumor collection was synchronized by staggering the timing of tumor implantation. Specifically, mice designated for the residual disease group were implanted ≈10 days earlier than those in the regressing group, enabling simultaneous tissue harvest after different durations of BRAF/MEKi treatment (14 days for residual disease, 3 days for regression). This design ensured that both tumor types were collected and processed on the same experimental day.

Single‐cell suspensions were obtained, followed by melanoma cell and fibroblast enrichment and RNA extraction as above. RNA sample quality was confirmed using a Qubit RNA HS Assay Kit (Invitrogen, catalog no. Q25855) to determine the concentration and with a Fragment Analyzer (Agilent) to determine RNA integrity. PolyA+ RNA was isolated with the NEBNext Poly(A) mRNA Magnetic Isolation Module (New England Biolabs, catalog no. E7490L). For Illumina library preparation, TruSeq‐barcoded RNAseq libraries were generated with the NEBNext Ultra II Directional RNA Library Prep Kit (New England Biolabs, catalog no. E7760L). Each library was quantified with a Qubit 1X dsDNA HS Assay Kit (Invitrogen, catalog no. Q33231) and the size distribution was determined with a Fragment Analyzer (Agilent) prior to pooling. Libraries were sequenced on an Illumina instrument. At least 20 m reads were generated per library. After sequencing, the reads were trimmed for low‐quality and adaptor sequences with TrimGalore v0.6.0, a wrapper for cutadapt and fastQC. Unwanted reads were removed with STAR v2.7.0e. Reads were subsequently mapped to the mouse transcriptome (Ensembl GRCm38) using STAR v2.7.0e. SARTools and DESeq2 v1.26.0 were used to generate normalized counts and statistical analysis of differential gene expression.

### Immunofluorescence (IF) Staining

Tumors were collected and embedded in an optimal cutting temperature (OCT) compound (Thermo Fisher Scientific, catalog no. 23730571), snap‐frozen, and stored at −80 °C. For IF staining of frozen tissue, 8‐µm sections of tumors were cut on a cryostat, fixed in 10% neutral‐buffered formalin for 10 min, followed by two washes of distilled water for 10 min each. Subsequently, each section was blocked for nonspecific protein interactions with 10% donkey serum in PBST, followed by staining of primary antibodies overnight at 4 °C (Table S4, Supporting Information). The following day, slides were washed with PBST and incubated for 1 h at RT with secondary antibodies conjugated to fluorochromes. Fluoroshield mounting medium with DAPI (Abcam, catalog no. ab104139) was used for nuclei staining. Images were captured using a Leica DM2500 upright microscope with a DFC7000T camera. Quantification of Col1a1 fluorescent images was determined by dividing the positively stained area by the total area using CellProfiler (RRID:SCR_007358). Quantification of low‐ and high‐density collagen “pocket” areas and CD8⁺ T cell number was performed using Fiji (RRID:SCR_002285). Regions of interest (ROIs) were manually selected based on morphology and analyzed for percentage of Col1a1⁺ pixel area. Low‐density collagen was defined as <15% Col1a1⁺, and high‐density as >30%. “Pockets” were defined as compact, enclosed regions with <5% Col1a1⁺ signal. Quantifications were performed by an experimenter blinded to treatment conditions. To assist interpretation, representative pocket regions are outlined in Figure 6D.

### Picrosirius Red Staining

Tumors were collected and formalin‐fixed before paraffin embedding. Paraffin‐embedded tissues were cut into 5‐µm sections, deparaffinized and rehydrated. Sections were stained with Sirius Red (0.1% solution in saturated picric acid) (Electron Microscopy Sciences, catalog no. 26357‐02) for 1 h at room temperature, followed by two distilled water washes. Stained sections were dehydrated and mounted with Entellan mounting medium (Merck Millipore, catalog no. 1079610100). Stained slides were imaged by using an Aperio ScanScope CS pathology slide scanner (Aperio Technologies Inc, Vista, CA) and analyzed with a positive pixel algorithm (Aperio Image Analysis, version 9.0, Aperio Technologies Inc, Vista, CA) following the manufacturer's instructions (Aperio Image Analysis user's guide. Vista, Calif: Aperio Technologies Inc, 2008). After the hue value was set, the input parameters for hue range and color saturation threshold were fine‐tuned empirically for each slide by an operator who was not aware of the treatment groups. Software default intensity thresholds for weak, medium, and strong signals were maintained. Fine‐tuning was accomplished by the use of a markup image and visual inspection that confirmed the inclusion of all magenta‐stained fibers and the exclusion of negative fibers. The average intensity and number of positive (P), strong‐positive (SP), weak‐positive (WP), and negative pixels (NP) were determined and the proportion of collagen per tumor was determined as (WP+P+SP)/(WP+P+SP+NP) x100. For fiber thickness and maturity analysis, picrosirius red‐stained sections were examined on an Olympus BX41 microscope (Olympus America, Inc., Center Valley, PA). Circularly polarized light was obtained with an Olympus U‐GAN analyzer (upper filter) aligned at 45° to the fast axis of a quarter wave plate placed below. Images were captured with an Olympus DP28 digital camera and cellSens Entry 4.2 software at a final magnification of 100X using identical camera settings for all photomicrographs. Quantification was performed using FIJI (version 1.51) and a macro (PS RED QUANTIFICATION V1.1) by an operator who was not aware of the treatment groups.

### Masson's Trichrome Staining

Tumors were collected and embedded in an optimal cutting temperature (OCT) compound (Thermo Fisher Scientific, catalog no. 23730571), snap‐frozen, and stored at −80 °C. For Masson's trichrome staining of frozen tissue using Masson's Trichrome Stain Kit (Polysciences, catalog no. 25088–100), 8‐µm sections of tumors were cut on a cryostat, fixed in 10% neutral‐buffered formalin for 60 min, followed by incubation in Bouin's Fixative overnight at room temperature. The following day, sections were gently washed in running tap water to remove the picric acid for 5 min and rinsed in distilled water. Subsequently, slides were transferred to Phosphotungstic/phosphomolybdic acid for 10 min. After discarding the solution, slides were drained and transferred to Aniline Blue for 5 min, followed by three rinses in distilled water. Then the slides were transferred to 1% Acetic acid for 1 min and rinsed in distilled water. Slides were dehydrated, cleared in xylene, and mounted with a toluene‐based mounting medium. Quantification of blue collagen trichrome signal was determined by dividing the positively stained area by the total area using CellProfiler (RRID:SCR_007358).

### Flow Cytometry

Single‐cell suspensions from all samples were prepared with a 70‐µm cell strainer as above. Cells were counted by a Scepter 3.0 Handheld Automated Cell Counter (Millipore Sigma, catalog no. PHCC340KIT). Cell suspensions were incubated with an anti‐CD16/32 Fc receptor blocker (Biolegend, catalog no. 156603) and surface antibodies (Table S4, Supporting Information) for 30 min at 4 °C. For intracellular staining of S100B, cells stained with surface markers were fixed and permeabilized using the Intracellular Fixation & Permeabilization Buffer Set (ThermoFisher, catalog no. 88‐8824‐00) according to the manual's instructions, followed by anti‐S100B antibody staining. Dead cells were excluded by staining with fixable viability dye. Data were acquired on a BD Symphony flow cytometer. Compensation, analysis, and visualization of the flow cytometry data were performed using FlowJo Software (RRID:SCR_008520).

### Wnt Signaling Pathway Inhibition

To inhibit Wnt signaling, mice were administered WNT‐C59 (10 mg kg^−1^) (Medchem Express, catalog no. HY‐ 15659) while the control mice were administered with the equivalent volume of DMSO. WNT‐C59 was initially dissolved in DMSO and subsequently diluted into carboxymethylcellulose (CMC; 0.5% w/v), Tween 80 (0.05% v/v) in sterile PBS to a final volume of 200 µL and administered to mice daily by oral gavage for the duration of the study, starting from day 10 of BRAF/MEKi treatment.

### Alteration of ECM Organization

To alter ECM organization in and around the tumor, mice were administered DHB (40 mg kg^−1^) (Sigma–Aldrich, catalog no. 37580) or BAPN (100 mg kg^−1^) (Sigma, catalog no. A3134) in 100 µL of sterile PBS, while the control mice were administered with the equivalent volume of sterile PBS. These agents were injected intraperitoneally daily for the duration of the study, starting from day 10 of BRAF/MEKi treatment.

### CD8^+^ T Cell Depletion

For CD8^+^ T cell depletion, mice were intraperitoneally injected with an anti‐CD8b antibody (100 µg) (clone Ly‐3.2, BioXcell, catalog no. BE0223) 3 days and 1 day prior to DHB treatment, which continued once a week until tumor collection. Control mice were injected with an equivalent amount of IgG1 isotype control (BioXcell, catalog no. BE0088).

### NK Cell Depletion

For NK cell depletion, mice were intraperitoneally injected with an anti‐ASGM1 antibody (Wako Pure Chemicals, catalog no. 986–10001) (20 µL) in sterile PBS 2 days prior to DHB treatment, which continued every four days until tumor collection. Control mice were injected with an equivalent amount of rabbit serum control in PBS.

### Study Approval

All mouse protocols and experiments were approved by the IACUC at Cornell University under protocol 2016‐0034.

### Statistical Analysis

Graphs and statistical analysis were performed using GraphPad Prism 10 (RRID:SCR_002798) and RStudio (RRID:SCR_000432). Graphs show means ± SEM or means ± SD as annotated in the corresponding figure legend. The number of tumors per group used in each experiment was annotated in the corresponding figure legend as n. Two‐tailed Student *t*‐tests were used when two groups were compared with each other. A one‐way ANOVA test with multiple comparisons was used when comparing three or more groups. A *p* value of less than 0.05 was considered significant.

## Conflict of Interest

The authors declare no conflict of interest.

## Author Contributions

C.H conceived and conceptualized the study, designed and conducted experiments, analyzed and interpreted the data, and wrote the manuscript. J.C, L.R.D, and D.K conducted experiments. K.‐J. L, D.K, and S.P.M analyzed the data. A.C.W conceived and conceptualized the study, reviewed and edited the manuscript, and provided funding.

## Supporting information



Supporting Information

Supplementary Table 1

## Data Availability

The data that support the findings of this study are available from the corresponding author upon reasonable request.
