# Supplementary material for: Therapy‐Induced ECM Remodeling Creates a Transient Immune Barrier in Residual Melanoma
Source: Adv Sci (Weinh). 2025 Aug 22;12(42):e08451. doi: 10.1002/advs.202508451 (PMC12622497; doi:10.1002/advs.202508451)
Supplement: Supplementary file 1 — Supporting Information [file ADVS-12-e08451-s001.pdf]

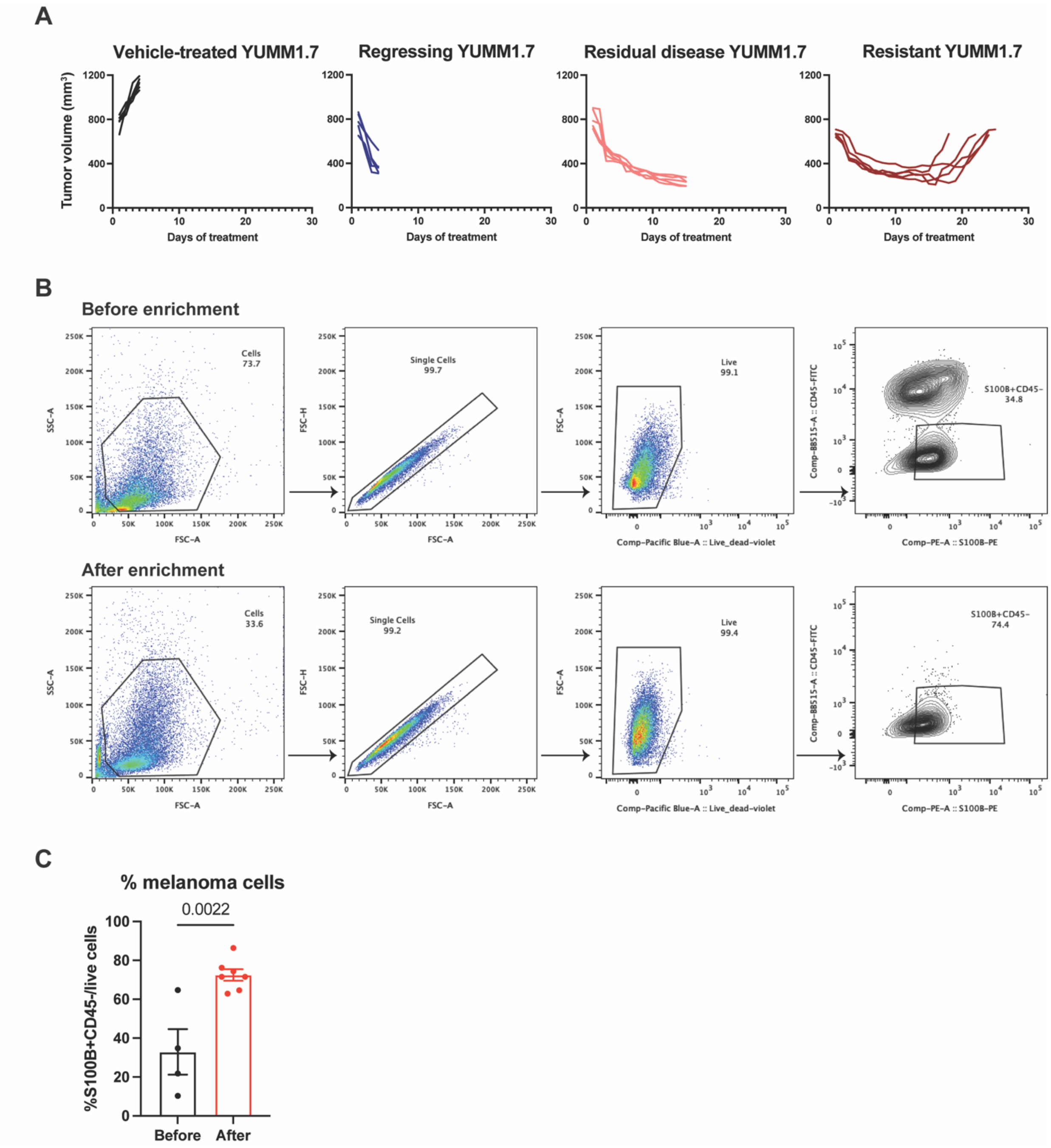

Figure S1. **YUMM1.7 melanoma mouse model and gating strategy for enriched melanoma cells for bulk mRNA-seq.** (**A**) Tumor volume curves of individual YUMM1.7 tumors during different phases. Vehicle-treated (growing) tumors were treated with 3 days of DMSO. Regressing tumors were treated with 3 days of BRAF/MEKi. Residual disease had been treated with 14 days of BRAF/MEKi. Resistant tumors were treated with BRAF/MEKi until tumors reached the initial size before treatment. **(B)** Flow cytometry gating strategy for melanoma cells from BRAF/MEKi-treated tumors. Melanoma cells are defined as CD45-S100B+ cells. **(C)** Quantification of CD45-

S100B+ melanoma cells before and after negative selection. Data presented as mean with SEM. Statistics were calculated using a two-tailed unpaired t-test.

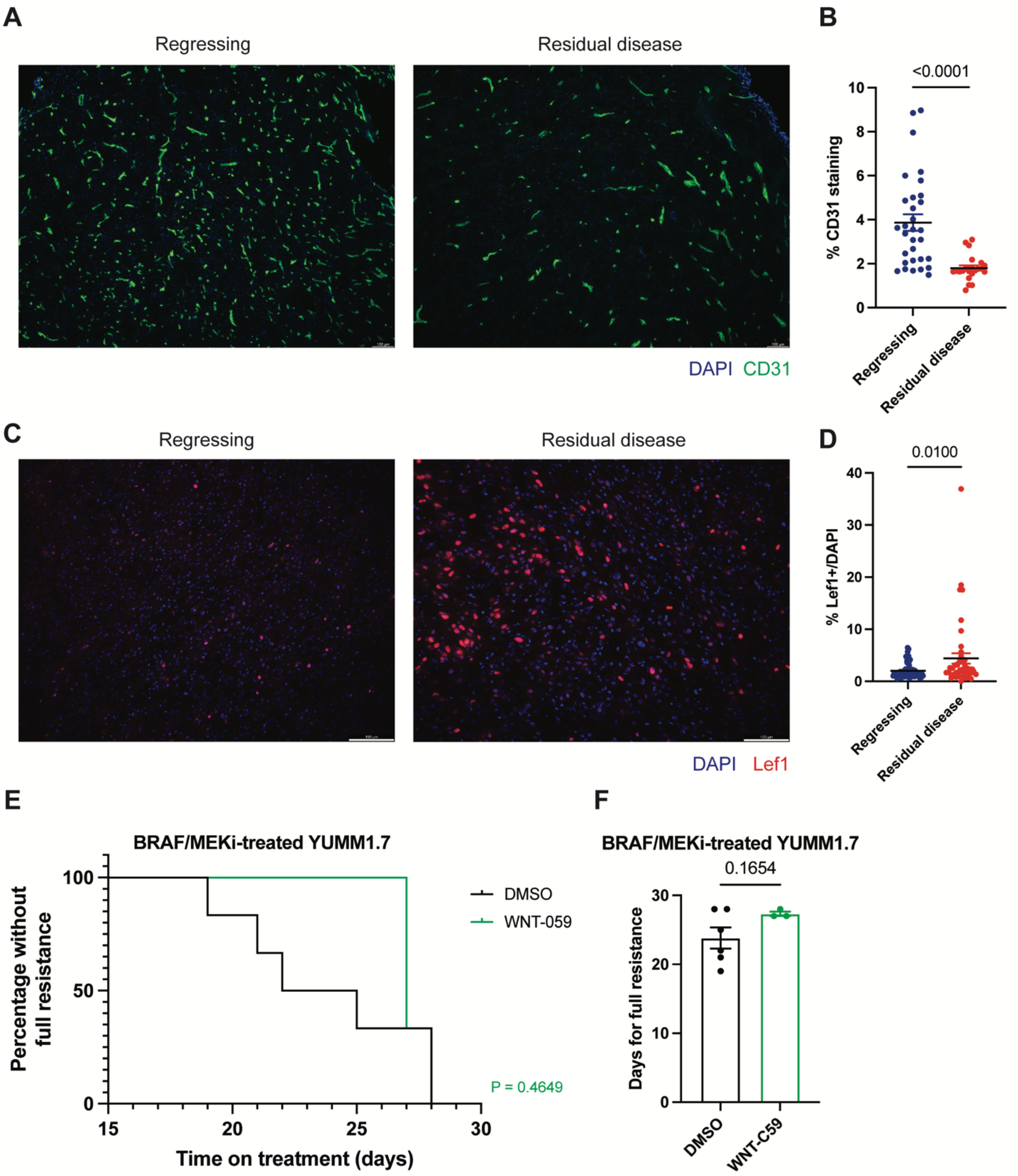

Figure S2. Evaluation of angiogenesis and Wnt signaling pathways in regressing versus residual disease tumors. **(A)** Representative immunofluorescence images of CD31 (green) and nuclear DAPI (blue) in regressing and residual disease tumors treated with BRAF/MEKi. Scale bar, 100 µm. **(B)** Quantification of percent CD31 area per field of melanoma tissue. n = 3 tumors per group, with 31 (regressing tumors) and 22 (residual disease) total microscopy fields analyzed across all tissues in respective groups. Data presented as mean with SEM. Statistics were

calculated using a two-tailed unpaired t-test. **(C)** Representative immunofluorescence images of Lef1 (red) and nuclear DAPI (blue) in regressing and residual disease tumors treated with BRAF/MEKi. Scale bar, 100 μm. **(D)** Quantification of the percentage of Lef1-positive cells per DAPI-positive cells. n = 3 tumors per group, with 58 (regressing tumors) and 45 (residual disease) total microscopy fields analyzed across all tissues in respective groups. Data presented as mean with SEM. Statistics were calculated using a two-tailed unpaired t-test. **(E)** Kaplan-Meier curve for BRAF/MEKi-treated mice bearing YUMM1.7 receiving WNT-C59, or DMSO. n = 3 and 6 tumors in the WNT-C59 and DMSO groups, respectively. Statistics were calculated using the Log-rank (Mantel-Cox) test. **(F)** Days for BRAF/MEKi-treated mice with WNT-C59, or DMSO to develop full resistance (tumors reached the initial size before BRAF/MEKi treatment, i.e. ~700mm^3 in size). n = 3, and 6 tumors in the WNT-C59 and DMSO groups, respectively. Data presented as mean with SEM. Statistics were calculated using a two-tailed unpaired t-test.

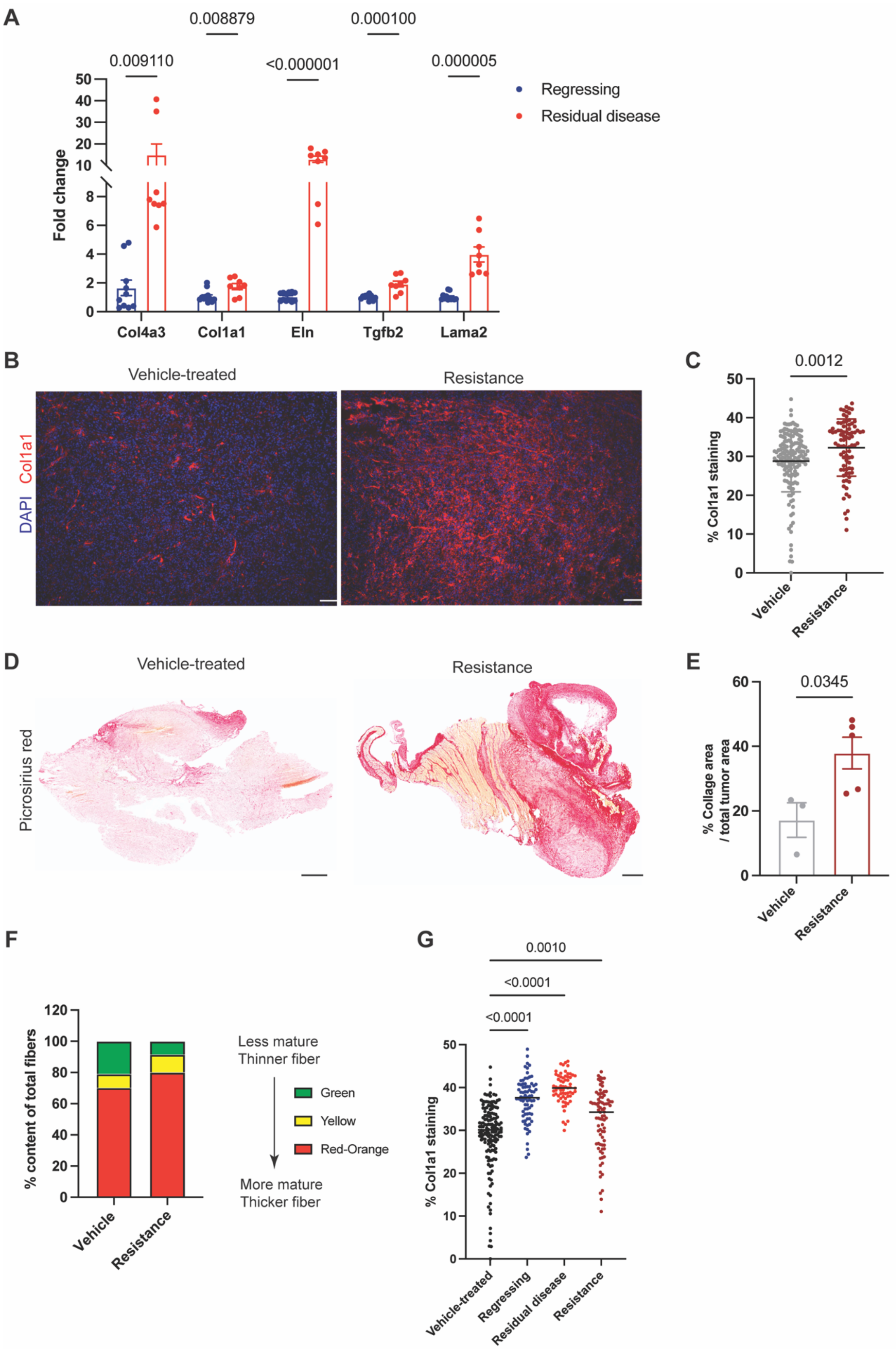
A
0.009110
0.008879
<0.000001
0.000100
0.000005
Fold change
Regressing
Residual disease
Col4a3
Col1a1
Eln
Tgfb2
Lama2
B
Vehicle-treated
Resistance
DAPI Col1a1
C
0.0012
% Col1a1 staining
Vehicle
Resistance
D
Vehicle-treated
Resistance
Picrosirius red
E
0.0345
% Collage area / total tumor area
Vehicle
Resistance
F
% content of total fibers
Vehicle
Resistance
Less mature
Thinner fiber
Green
Yellow
Red-Orange
More mature
Thicker fiber
G
0.0010
<0.0001
<0.0001
% Col1a1 staining
Vehicle-treated
Regressing
Residual disease
Resistance

Figure S3. ECM components are upregulated in BRAF/MEKi-tolerant tumors, and upregulation is maintained as tumors rebound. (A) Fold change of mRNA expression of selective ECM-related genes in regressing tumors and residual disease. β-Actin expression was used as a loading control. n = 6 and 4 in regressing tumors and residual disease, respectively. Technical replicates were performed with each sample. Data presented as mean with SEM. Statistics were calculated using multiple unpaired t-tests. **(B)** Representative immunofluorescence images of collagen 1a (Col1a1, red) and nuclear DAPI (blue) in growing and resistant tumors treated with BRAF/MEKi. Scale bar, 100μm. **(C)** Quantification of percent Col1a1 area per field of melanoma tissues. n = 6 tumors per group, with 152 (vehicle-treated tumors) and 82 (resistant tumors) total microscopy fields analyzed across all tissues in respective groups. Data presented as mean with SD. Statistics were calculated using a two-tailed unpaired t-test. **(D)** Representative picrosirius red staining showing collagen deposition, imaged using a ScanScope in growing tumors and resistant tumors treated with BRAF/MEKi. Scale bar, 500μm. **(E)** Quantification of percent collagen area per total tumor area. n = 3-5 tumors per group. Resistant tumors were from the same experiments as Fig. 3G (PBS, BRAF/MEKi-treated group). Data presented as mean with SEM. Statistics were calculated using a two-tailed unpaired t-test. **(F)** Quantification of collagen maturity and fiber thickness in growing and resistant tumors stained with picrosirius red using circular polarized light microscopy. Birefringence hue was quantified as a percent of total fibers. n = 3-5 tumors per group, 2-6 fields per tumor section. Resistant tumors were from the same experiments as Fig. 3G (PBS, BRAF/MEKi-treated group). Data presented as mean. **(G)** Quantification of percent Col1a1 area per field across melanoma tumors from different treatment phases: vehicle-treated, regressing, residual disease, and resistant tumors. Data are pooled from Figures 2F and S3C. Data presented as mean with SD. Statistics were calculated using ordinary one-way ANOVA.

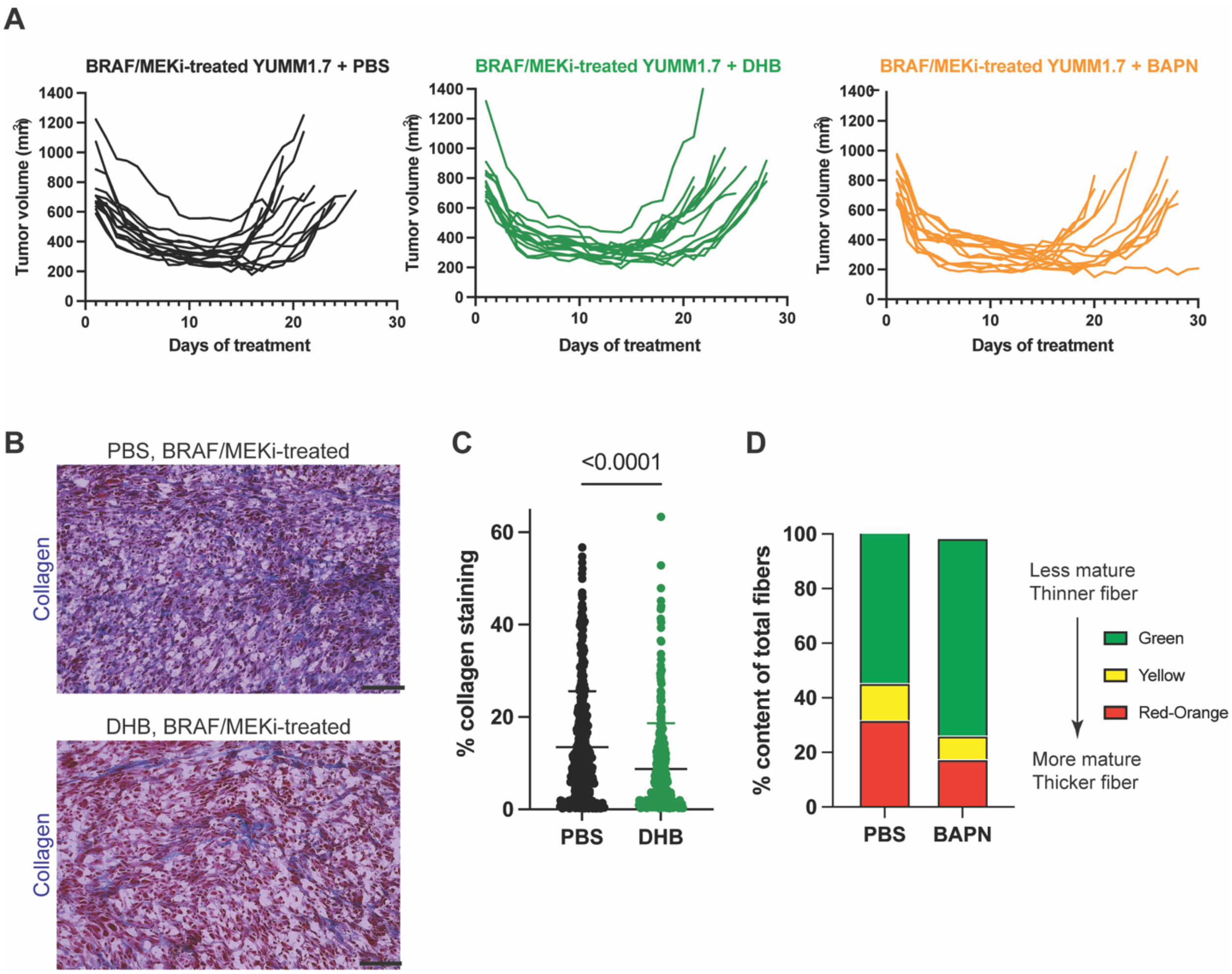

Figure S4. **Enzymatic inhibition in BRAF/MEKi-treated tumors decreases collagen deposition and delays the onset of therapy resistance.** (**A**) Tumor volume curves of individual BRAF/MEKi-treated tumors with injection of PBS, DHB, or BAPN. **(B)** Representative trichrome stains of collagen (blue) of BRAF/MEKi-treated tumors in the indicated treatment groups. Scale bar, 100µm. **(C)** Quantification of percent collagen area per field of melanoma tissues. n = 6 tumors per group, with 438 (PBS) and 316 (DHB) total microscopy fields analyzed across all tissues in respective groups. Data presented as mean with SD. Statistics were calculated using a two-tailed unpaired t-test. **(D)** Quantification of collagen maturity and fiber thickness in PBS- and BAPN-treated tumors stained with picrosirius red using circular polarized light microscopy. Birefringence hue was quantified as a percent of total fibers. n = 4 tumors per group, 3-7 fields per tumor section. Data presented as mean.

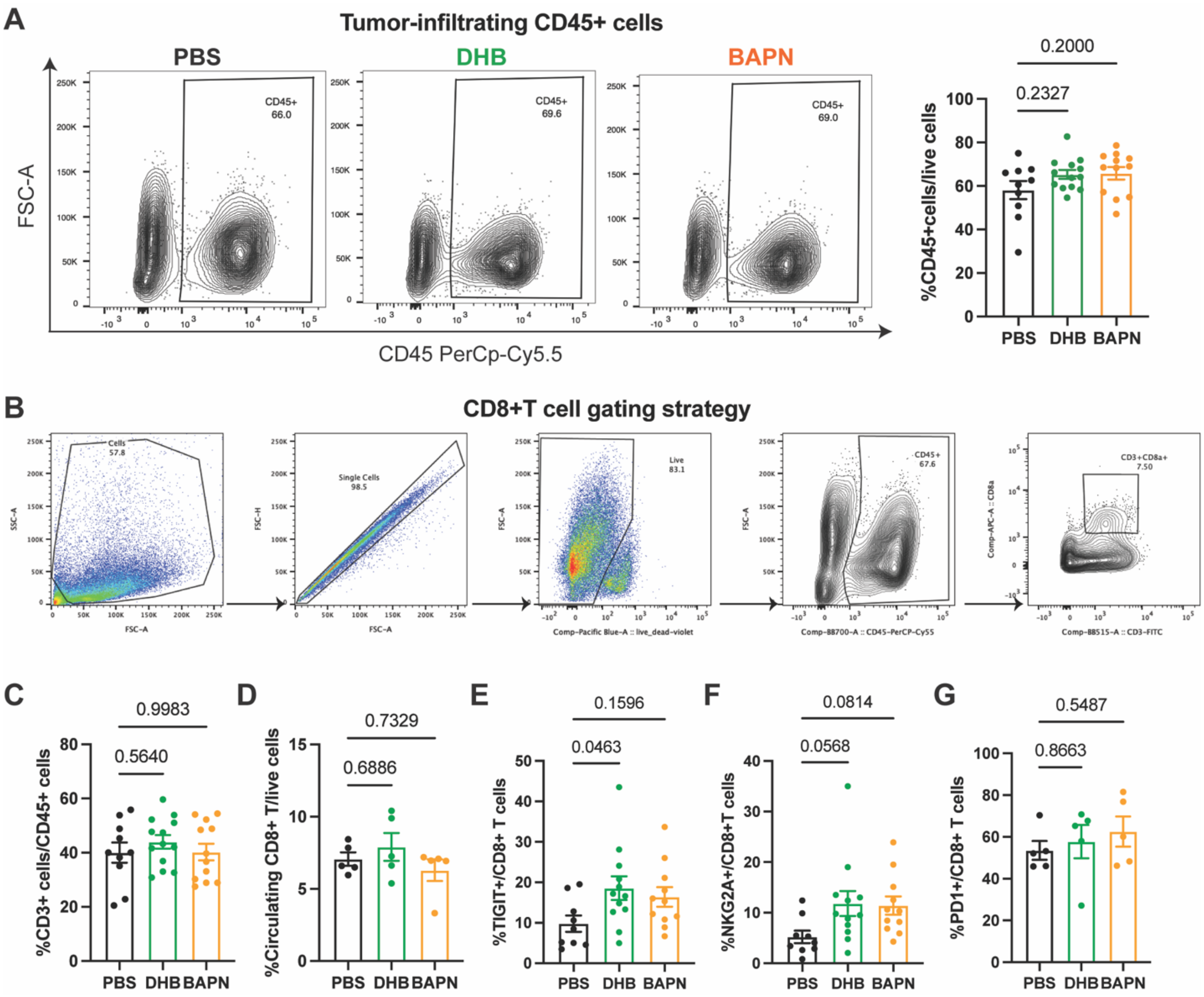

Figure S5. **Targeting the ECM enhances CD8+ T cell-anti-tumor immunity.** (**A**) Flow cytometry analysis of tumor-infiltrating CD45+ cells gated on live cells in BRAF/MEKi-treated mice injected intraperitoneally with PBS, DHB or BAPN. Left, representative flow cytometry plots. Numbers on the plots represent the percentage of cells within each gate. Right, quantification of tumor-infiltrating CD45+ cells in each group. n = 10, 13, and 12 tumors in the PBS, DHB and BAPN groups, respectively. Data presented as mean with SEM. Statistics were calculated using one-way ANOVA with Dunnett's multiple comparisons test. **(B)** Flow cytometry gating strategy for tumor-infiltrating CD8+ T cells from BRAF/MEKi-treated mice injected intraperitoneally with PBS, DHB or BAPN. **(C)** Flow cytometry quantification of $CD3^{+}$ T cells among $CD45^{+}$ leukocytes in BRAF/MEKi-treated mice injected intraperitoneally with PBS, DHB, or BAPN. n = 10, 13, and 12 tumors in the PBS, DHB, and BAPN groups, respectively. Data presented as mean with SEM. Statistics were calculated using one-way ANOVA with Dunnett's multiple comparisons test. **(D)** Flow cytometry quantification of circulating $CD8^{+}$ T cells among live peripheral blood cells in BRAF/MEKi-treated mice injected with PBS, DHB, or BAPN. n = 5 mice in each group. Data presented as mean with SEM.

Statistics were calculated using one-way ANOVA with Dunnett's multiple comparisons test. **(E)** Flow cytometry quantification of TIGIT$^+$ cells among tumor-infiltrating CD8$^+$ T cells in BRAF/MEKi-treated mice injected with PBS, DHB, or BAPN. n = 9, 12, and 11 tumors in the PBS, DHB, and BAPN groups, respectively. Data presented as mean with SEM. Statistics were calculated using one-way ANOVA with Dunnett's multiple comparisons test. **(F)** Flow cytometry quantification of NKG2A$^+$ cells among tumor-infiltrating CD8$^+$ T cells in BRAF/MEKi-treated mice injected with PBS, DHB, or BAPN. n = 9, 12, and 11 tumors in the PBS, DHB, and BAPN groups, respectively. Data presented as mean with SEM. Statistics were calculated using one-way ANOVA with Dunnett's multiple comparisons test. **(G)** Flow cytometry quantification of PD-1$^+$ cells among tumor-infiltrating CD8$^+$ T cells in BRAF/MEKi-treated mice injected with PBS, DHB, or BAPN. n = 5 tumors in each group. Data presented as mean with SEM. Statistics were calculated using one-way ANOVA with Dunnett's multiple comparisons test.

.

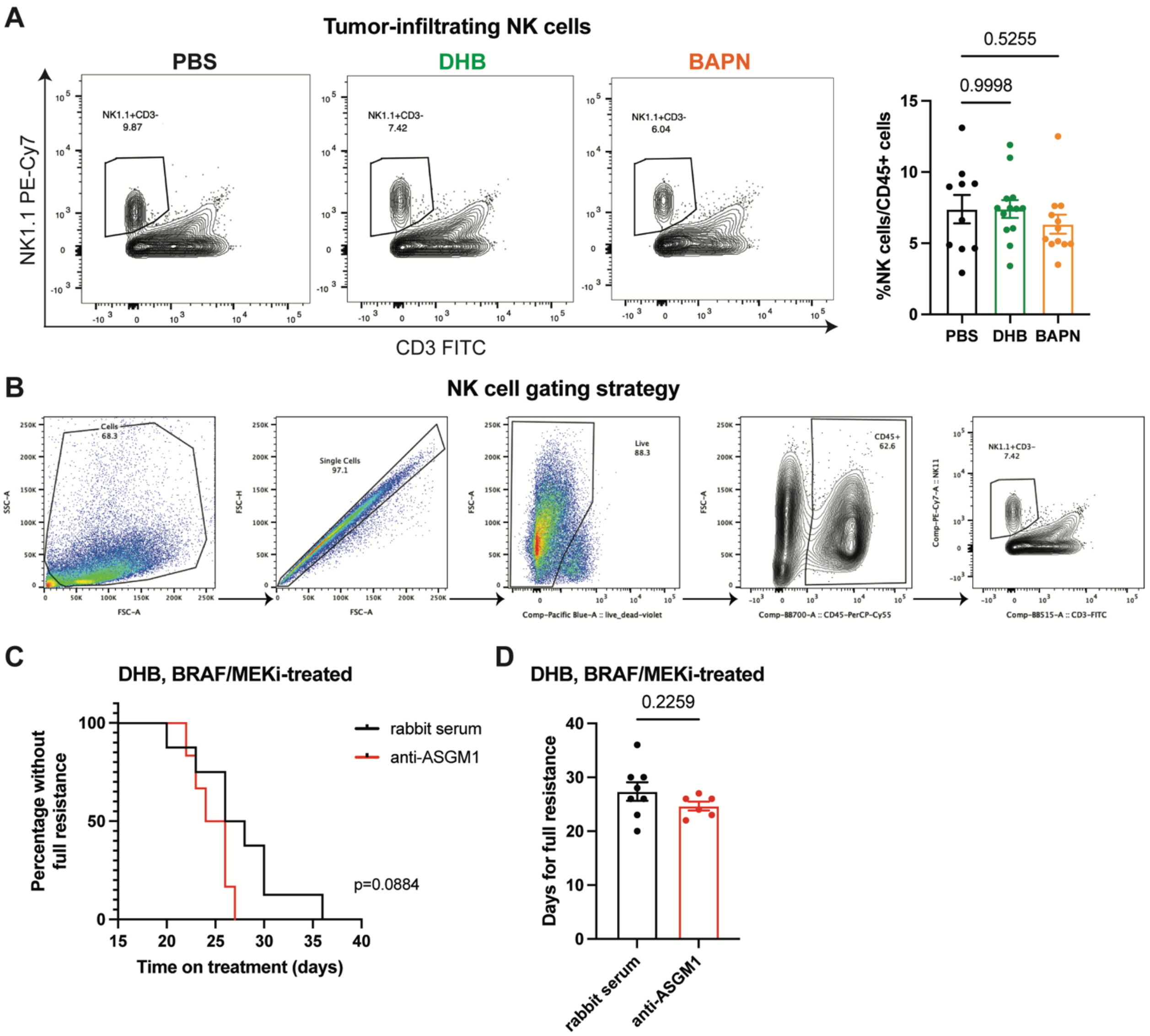

Figure S6. **NK cell infiltration and depletion do not impact the therapeutic benefit of ECM inhibition. (A)** Flow cytometry analysis of tumor-infiltrating NK cells gated on live/CD45+ cells in BRAF/MEKi-treated mice injected intraperitoneally with PBS, DHB or BAPN. Left, representative flow cytometry plots. Numbers on the plots represent the percentage of cells within each gate. Right, quantification of tumor-infiltrating NK cells in each group. n = 10, 13, and 12 tumors in the PBS, DHB and BAPN groups, respectively. Data presented as mean with SEM. Statistics were calculated using one-way ANOVA with Dunnett's multiple comparisons test. **(B)** Flow cytometry gating strategy for tumor-infiltrating NK cells from BRAF/MEKi-treated mice injected intraperitoneally with PBS, DHB or BAPN. **(C)** Kaplan-Meier curve for BRAF/MEKi + DHB-treated mice bearing YUMM1.7 receiving anti-ASGM1 antibodies or rabbit serum control. Statistics were calculated using the Log-rank (Mantel-Cox) test. **(D)** Days for BRAF/MEKi + DHB-treated mice with anti-ASGM1 antibodies or rabbit serum control to develop full resistance (tumors reached the initial size before BRAF/MEKi treatment, i.e. ~700mm^3 in size). n = 8 and 6

tumors in the anti-ASGM1 and rabbit serum control groups, respectively. Data presented as mean with SEM. Statistics were calculated using a two-tailed unpaired t-test.

Table S2. Summary of treatment regimens and clinical characteristics for patient samples in GSE75299 and GSE50535

GSE75299				
**Patient**	**Treatment (mg)**	**Biopsy timing**	**Best overall response**	**Progression free survival (days)**
1	vemurafenib 960 bid	Baseline and Day 85	-32%	258
3	vemurafenib 960 bid cobimetinib 60 qd	Baseline and Day 22	-46%	145
4	vemurafenib 960 bid cobimetinib 60	Baseline and Day 261	-63%	+3 years
6	trametinib 2 qd	Baseline and Day 15	>-30%	62
7	vemurafenib 960 bid trametinib 2 qd	Baseline and Day 12	<-30%	300
8	dabrafenib 150 bid trametinib 2 qd	Baseline and Day 22	>-30%	50

bid, twice a day; qd, once daily

GSE50535				
**Patient**	**Treatment (initiation date)**	**Date of biopsy**	**Response at biopsy**	**Time to progression**
2	Vemurafenib (3/13/12)	2/9/12 and 6/25/12	PD	4 months
3	Dabrafenib (7/29/11)	9/8/10 and 5/24/12	PD	10 months
5	Trametinib (3/28/11)	3/28/11 and 10/24/11	PD	<7 months

PD, Progressive Disease

Table S3. qPCR primers

Gene name	Assay name (IDT Technologies)	Sequence
Col1a1	Mm.PT.58.7562513	Primer 1: 5-CGCAAAGAGTCTACATGTCTAGG-3'<br>Primer 2: 5-CATTGTGTATGCAGCTGACTTC-3'
Col4a3	Mm.PT.58.9846683	Primer 1: 5-GGACTGGGTTTCTCTCTGGA-3'<br>Primer 2: 5-CTATAAATGGACTGGCTCGGAA-3'
Eln	Mm.PT.58.43682390	Primer 1: 5-AGTTCCTGGTGTTGGTCTTC-3'<br>Primer 2: 5'-CCTTGGCTTTGACTCCTGT-3'
Tgfb2	Mm.PT.58.14105470	Primer 1: 5'-TGTACCTTCGTGCCGTCTA-3'<br>Primer 2: 5-CTGATCACCACTGGCATATGTAG-3'
Lama2	Mm.PT.58.31 899903	Primer 1: 5'-CTCCAGCCAAACCATCAGTC-3'<br>Primer 2: 5'-TTCCTAACACAGCCATCCAG-3'
Actb	Mm.PT.39a.22214843.g	Primer 1: 5'-GATTACTGCTCTGGCTCCTAG-3'<br>Primer 2: 5'-GACTCATCGTACTCCTGCTTG-3'

Table S4. Antibodies

Antigen	Clone	Vendor	Catalog Number	Application	Dilution
Col1a1	E8F4L	Cell Signaling	72026T	IF	1:100
CD45	30-F11	Biolegend	103101	IF	1:800
CD3	17A2	Biolegend	100201	IF	1:800
CD31	MEC13.3	Biolegend	102501	IF	1:200
CD8a	208	Sino Biological	50389-R208	IF	1:100
Lef1	C12A5	Cell Signaling	2230	IF	1:200
CD45-FITC	30-F11	ThermoFisher	11-0451-81	FC	0.5 µg/test
CD45-PerCp-Cy5.5	30-F11	Biolegend	103132	FC	0.25 µg/test
CD3-FITC	17A2	Biolegend	100204	FC	1.0 µg/test
CD8a-APC	53-6.7	Biolegend	100712	FC	0.25 µg/test
NK1.1-PE-Cy7	PK136	ThermoFisher	25-5941-81	FC	0.25 µg/test
NKG2A-PE	16A11	Biolegend	142803	FC	0.5 µg/test
PD-1-PE	29F.1A12	Biolegend	135205	FC	1.0 µg/test
S100B-PE	15F9NB	Novus Biologicals	NBP2-45267PE	FC	2.5 µg/ml
TIGIT	1G9	Biolegend	142109	FC	0.5 µg/test
